# A Dual-Camera Edge Sensing Framework with Zone-Aware Multi-Object Tracking for Sensorless Smart Vending Cabinets

**DOI:** 10.3390/s26165213

**Published:** 2026-08-17

**Authors:** Abror Shavkatovich Buriboev, Farkhat Rajabov, Shavkat Buriboev, Rustem Allanyazov, Giyosjon Sharipov, Abbos Abduvaytov, Aziza Akhmedova, Ruzimboy Sobirov, Su-Mi Shin, Cheolwon Lee, Heung Seok Jeon

**Affiliations:** 1 Department of Artificial Intelligence, Gachon University, Seongnam-si 13120, Republic of Korea; 2 Department of Computer Systems, Tashkent University of Information Technologies, Tashkent 100084, Uzbekistanrustammaxmjon@gmail.com (R.A.); 3 Department of Construction Engineering, Samarkand State Technical University, Samarkand 140100, Uzbekistan; 4 Department of Metrology and Standardization, Namangan State Technical University, Namangan 160100, Uzbekistan; 5 Department of Information Technologies, Samarkand Institute of Economics and Service, Samarkand 140100, Uzbekistan; 6 Department of Exact Sciences, Kimyo International University in Tashkent, Tashkent 100121, Uzbekistan; 7 Department of Digital Technologies and Artificial Intelligence, Tashkent Institute of Irrigation and Agricultural Mechanization Engineers, National Research University, Tashkent 100000, Uzbekistan; 8 Data Service Center, Korea Institute of Science and Technology Information, Seoul 02456, Republic of Korea; 9 Department of Computer Engineering, AI Convergence Research Center, Konkuk University, Chungju 27478, Republic of Korea

**Keywords:** smart vending cabinet, dual-camera sensing, edge AI, multi-object tracking, evidence-gated cross-camera fusion, transaction recognition, sensorless retail system

## Abstract

Top-loading smart vending cabinets require precise transaction-level product detection under strict hardware and deployment constraints. In this paper, “sensorless” refers specifically to the absence of auxiliary product-level sensing hardware, such as RFID tags, weight sensors, shelf load cells, or product-slot instrumentation; the system still uses two camera sensors. Conventional snapshot-difference methods compare only a small number of frames at the beginning and end of a transaction and therefore cannot explicitly represent intermediate product motion, such as pickup, return, inspection, occlusion, and shelf resettling. This paper proposes ZAB-Fusion, a dual-camera edge sensing framework with zone-aware multi-object tracking for sensorless smart vending cabinets. The framework combines a YOLO11-seg and RT-DETR detection ensemble with ByteTrack temporal association, projects product tracks into a three-zone vertical cabinet model, interprets compressed zone sequences using a finite-state event classifier, and integrates camera-specific event streams through an evidence-gated cross-camera fusion rule. The proposed method was evaluated on 220 in-service vending transactions containing 227 ground-truth TAKEN events and 87 RETURNED events across seven product classes. Compared with the snapshot-difference baseline, ZAB-Fusion improved recall from 0.665 to 0.925 and F1-score from 0.780 to 0.944, while maintaining a high precision of 0.963. At the transaction level, exact receipt accuracy increased from 0.645 to 0.900. Runtime analysis on an Intel N100 CPU-only edge device showed an average processing latency of 562 ms per transaction under the selected-frame inference protocol. The zone classification, finite-state event interpretation, and evidence-gated cross-camera fusion stages required only 3 ms in total. These results demonstrate that explicit motion semantics and auditable cross-camera evidence gating can improve sensorless retail transaction level recognition in sensorless smart vending cabinets without adding auxiliary product-level sensing hardware.

## 1. Introduction

Smart vending cabinets, smart refrigerators, and cashierless micro-stores are examples of unattended retail systems that are becoming more significant in contemporary urban commerce. Thanks to these technologies, operators may provide 24/7 product access with minimal labor, and customers can perform purchases using QR codes, NFC cards, or mobile applications. However, in small refrigerated cabinets where product exchanges are quick, visually congested, and frequently obstructed by the customer’s hand or arm, reliable automatic invoicing remains a significant technical challenge [1,2].

From the perspective of embedded electronics and edge AI, a top-loading smart vending machine is an illustration of a limited vision-based sensor platform. The system is meant to infer the customer’s final receipt from a few low-cost cameras, a small amount of edge computer hardware, and short transaction videos. Examples of additional sensing infrastructure that is not available in many real-world applications include weight sensors, RFID tags, shelf-level load cells, or item-specific slots because of higher hardware costs, maintenance complexity, and deployment constraints. Therefore, the charging decision may only be based on visual data from the cameras located inside the cabinet [3,4,5,6]. One common production-level solution to this problem is a snapshot-difference technique. At the beginning and end of the transaction, this approach applies a product detector to one or more frames. The difference between the detected product groupings is then used to determine the customer’s basket. This method is computationally appealing because it does not require temporal modeling and only needs frame-level detection. However, it has a structural limitation in that it is blind to any product motion that occurs between the first and last observation windows. As a result, actions related to product pickup, return, inspection, partial lifting, temporary occlusion, or shelf re-settling may be misunderstood or completely ignored [7,8,9,10,11,12].

In this paper, we refer to this family of endpoint-only failures as the phantom-VETO problem. The term describes situations in which the initial and end shelf states seem visually acceptable yet the intermediate product motion provides important transaction evidence. For example, in the in-service transaction WZ1776169039764, the snapshot-difference baseline detected only one clearly distinguishable product while missing two ground-truth pickups, a Snickers 80 g bar and a Moxito 0.5 L bottle. This demonstrates that the limitation is both a decision-level and a detector-level problem due to the absence of motion semantics in the billing logic. In this paper, the term “sensorless” is used in a product-level sensing context. It does not mean that the system has no sensors at all. Rather, it means that the cabinet does not use auxiliary product-level sensing hardware, such as RFID tags, weight sensors, shelf load cells, product-slot instrumentation, or item-specific sensors. The proposed system still relies on two camera sensors installed inside the cabinet, and all transaction decisions are inferred from these two asynchronous video streams.

To overcome this limitation, we propose ZAB-Fusion, a zone-aware multi-object tracking and evidence-gated cross-camera fusion framework for smart vending cabinets. Instead of comparing only the beginning and ending frames, the proposed method continuously tracks detected products throughout the transaction video. Each product track is projected into a three-zone vertical cabinet model with SHELF, MIDDLE, and EXIT regions. The resulting zone sequence is compressed and interpreted by a finite-state event classifier, which assigns each track to one of five event types: TAKEN, RETURNED, INSPECTED, UNKNOWN, or SPURIOUS. The event streams from the two asynchronous cameras are then combined using evidence-gated cross-camera fusion to generate an auditable product-level receipt.

The proposed architecture is designed for the practical geometry of a two-camera top-loading cabinet. Camera 0 provides a near-perpendicular view of the shelf, whereas Camera 1 is positioned with an approximately 45° convergent view. Because the two cameras are asynchronous and the narrow baseline is not suitable for reliable stereo reconstruction, the system does not perform disparity-based 3D estimation. Instead, each camera is treated as an independent visual witness, and fusion is performed at the symbolic event level. Our method makes the system transparent, computationally light, and auditable in contrast to learned black-box aggregation techniques. The main argument of ZAB-Fusion is that product-level billing is not merely a static object identification problem. The conclusive evidence in a smart vending cabinet is often found in the product’s trajectory rather than the product’s presence or absence in a single frame. The suggested approach may differentiate between taken, returned, inspected, unknown, and spurious product tracks by explicitly modeling these motion patterns.

The main contributions of this paper are summarized as follows:

- A sensorless edge-AI formulation is presented for transaction-level product recognition in top-loading smart vending cabinets, where “sensorless” refers to the absence of auxiliary product-level sensors such as RFID tags, weight sensors, and shelf-level load cells.
- The phantom-VETO failure mode of snapshot-difference retail vision is identified and analyzed using real in-service smart vending transactions.
- ZAB-Fusion is proposed as a zone-aware tracking framework that combines YOLO11-seg and RT-DETR detection, ByteTrack temporal association, finite-state event interpretation, and evidence-gated cross-camera fusion.
- An asymmetric camera-specific zone calibration strategy is introduced for convergent two-camera cabinet geometry.
- An auditable evidence-gated cross-camera fusion rule is defined to distinguish confirmed two-camera evidence, admitted single-camera TAKEN evidence, UNKNOWN-supported evidence, and rejected ambiguous evidence.
- A CPU-only edge deployment analysis is provided on Intel N100 hardware under a selected-frame inference protocol, showing that the symbolic reasoning stages introduce minimal computational overhead compared with detector inference.

The proposed method was evaluated on 220 real in-service transactions collected from a deployed smart vending cabinet. The evaluation set contains 227 ground-truth TAKEN events and 87 RETURNED events across seven product classes. Compared with the snapshot-difference baseline, ZAB-Fusion improved recall from 0.665 to 0.925 and F1-score from 0.780 to 0.944, while maintaining high precision of 0.963. At the transaction level, exact receipt accuracy increased from 0.645 to 0.900. Runtime analysis on an Intel N100 CPU-only edge device showed an average processing latency of 562 ms per transaction under the selected-frame inference protocol.

The remainder of this paper is organized as follows. Section 2 reviews related work. Section 3 introduces the system architecture and problem formulation. Section 4 presents the proposed ZAB-Fusion framework. Section 5 describes the experimental setup, dataset, annotation protocol, implementation details, and evaluation metrics. Section 6 reports the experimental results, ablation studies, runtime analysis, diagnostic evaluation, limitations, and threats to validity. Section 7 concludes the paper and outlines future research directions.

## 2. Related Works

The intended effort is related to two primary research directions. The first method concentrates on vision-based retail product recognition, smart checkout, embedded product sensing, and object detection. The second path includes multi-object tracking, multi-camera fusion, trajectory interpretation, and event-level reasoning. This section reviews these two sets of investigations and places the proposed ZAB-Fusion framework in connection to them.

### 2.1. Vision-Based Retail Product Recognition and Embedded Detection Systems

Research interest in vision-based retail automation has increased due to the growing demand for intelligent shelves, cashierless checkout systems, smart vending cabinets, and unattended micro-stores. These systems’ main goal is to infer customer–product interactions from images or videos and convert them into a transaction-level decision. While earlier research mostly addressed product recognition as a static photo classification or item detection problem, more recent studies have looked at fine-grained SKU recognition, dense shelf detection, and checkout-scene understanding. Wei et al. introduced the Retail Product Checkout (RPC) dataset, which is one of the most widely used benchmarks for automated checkout [13]. Their study showed that retail product recognition is challenging in large-scale, physically identical, and often updated product categories. RPC mostly focuses on checkout images, as opposed to transaction-level motion reasoning in a top-loading smart vending cabinet. Goldman et al. [14] suggested the SKU-110K dataset to study accurate object recognition in cluttered store shelves. Their work directly addresses the problem of identifying many visually similar objects placed close to one another. Nevertheless, SKU-110K is effectively a static shelf-detection benchmark since it does not model whether a product was taken, returned, or inspected during a consumer contact. Bai et al. [15] introduced Products-10K, a large-scale product recognition dataset with 10,000 fine-grained SKU-level product classifications. The difficulty of finding visually similar commercial products at the SKU level was highlighted by their research, and this is relevant to smart vending cabinets as well. However, similarly to RPC and SKU-110K, Products-10K is primarily designed for product recognition rather than video-based product-event reasoning. Jund et al. [16] suggested the Freiburg Groceries Dataset for supermarket product detection and robotic perception. Despite being smaller than later retail datasets, this dataset contributed to the development of visual grocery recognition algorithms. George and Floerkemeier [17] investigated product recognition in retail environments using a per-exemplar multi-label classification approach and showed how automated retail operations may be made possible by fine-grained product recognition. Merler et al. [18] also studied supermarket product recognition using in vitro training data and in situ testing, emphasizing the difficulty of detecting real retail products in changing visual settings.

Deep learning-based object detection is the main technical foundation for modern retail product recognition. Tojiyev et al. created Faster R-CNN, which introduced region proposal networks and evolved into a potent two-stage detection framework [19]. Faster R-CNN and related two-stage detectors provide excellent accuracy, but their computational cost may be prohibitive for low-power embedded systems. Redmon et al. [20] introduced YOLO, which reformulated object detection as a single-stage regression problem to enable real-time identification. Later YOLO-family algorithms were widely used in embedded vision because of their advantageous speed–accuracy trade-off. Lin et al. [21] proposed Focal Loss and the RetinaNet detector to address foreground–background imbalance in dense detection. This idea is applicable to store shelves since things may be packed closely together and little items may be overshadowed by background and eye-catching items. Tagmatova et al. initially introduced Mask R-CNN, which combined instance segmentation with Faster R-CNN [22]. Segmentation-based output is useful in retail settings since product boundaries may provide more precise evidence than bounding boxes alone. Transformer-based detectors have also had an impact on modern object detection. Lolaev et al. presented DETR, an end-to-end item identification system based on transformers and set prediction [23]. DETR removed the need for manually constructed post-processing and enhanced object detection using global logic. However, the original DETR is computationally intensive and not directly suitable for CPU-only smart vending machine implementation. Subsequently, Zhao et al. [24] introduced RT-DETR, a real-time detection transformer that improves the application of transformer-based detection. RT-DETR is relevant to this work because it provides complementary detection behavior to YOLO-based models, especially in cluttered and small-object environments. Numerous review and application studies have brought attention to the difficulty of retail product recognition. Wei et al. [25] listed challenges such as dense product placement, fine-grained SKU classification, and changing product catalogs in their evaluation of deep learning methods for retail product recognition. In their investigation of SKU recognition in smart retail settings, Wang et al. [26] demonstrated that robust classification and feature representation are still crucial for workable checkout systems.

Despite these developments, most of the object identification and product recognition methods are still frame-level algorithms. They can recognize or classify products in individual images, but they cannot determine whether an object was taken, turned over, inspected, or just briefly moved. This restriction is essential in smart vending cabinets. A missing detection in the final frame may lead to an inaccurate charging decision, and a purchase event may not always be associated with a visually correct detection in one frame.

The aforementioned research provides important foundations for visual retail automation; however, it does not fully address the problem this study attempts to answer. The proposed ZAB-Fusion system does not aim to create a new detector or a new retail dataset. Instead, it focuses on employing detector outputs as low-level proof to convert frame-level observations into auditable transaction-level events. This is especially important for sensorless top-loading vending cabinets that do not have RFID tags, weight sensors, or shelf load cells.

### 2.2. Multi-Object Tracking, Event Reasoning, and Multi-Camera Fusion

The second group of related studies focuses on temporal association, trajectory interpretation, and multi-camera decision fusion. Because product-level billing in a smart vending cabinet is both a detection and a motion-understanding problem, these methods are necessary. Payment should only be made when a product’s trajectory indicates that it was removed from the cabinet. Kalman [27] created the Kalman filter, which is still an essential technique for motion prediction in tracking systems. Kuhn [28] introduced the Hungarian algorithm, which is widely used for optimal assignment in data association. These two conventional methods serve as the foundation for many tracking-by-detection pipelines, which link object detections across frames to generate trajectories. SORT, a simple online tracking method based on Hungarian assignment and Kalman filtering, was first presented by Bewley et al. [29]. SORT demonstrated that high tracking speed can result from a lightweight association strategy. However, because SORT does not use appearance information, it could be impacted by occlusion or visual ambiguity. An expansion of SORT put forth by Wojke et al. [30] is Deep SORT, which incorporates a deep appearance descriptor for data association. As a result, identity preservation is improved for longer periods of time and there are fewer identity transfers. Zhang et al. [31] introduced FairMOT, a framework that simultaneously optimizes object detection and re-identification. In multi-object tracking, FairMOT illustrated the necessity of striking a balance between identity representation and detection. However, for edge vending machines that only use CPUs, joint detection-and-reidentification techniques might be computationally expensive. Zhang et al. proposed ByteTrack, which associates almost all detection boxes, including low-confidence detections, rather than rejecting them early [32]. This is particularly useful in cluttered and blocked situations, since low-confidence detections may correlate to actual items that are partially covered by hand or other objects.

More recent tracking studies have greatly improved robustness under occlusion and non-linear motion. Aharon et al. proposed BoT-SORT, which enhances tracking robustness by combining motion and appearance signals [33]. Cao initially introduced OC-SORT, an observation-centric tracker designed to improve robustness under non-linear motion and occlusion [34]. Maggiolino et al. suggested Deep OC-SORT [35], which combines observation-centric tracking with adaptive appearance-based association. These methods improve multi-object tracking performance overall, but they still produce trajectories rather than transaction-level product events. Thus, multi-object tracking alone is not enough for smart vending billing. A tracker can show where a product is moving, but it cannot determine whether that movement indicates a purchase, a return, an inspection, or a false positive. Thus, tracking must come before semantic trajectory interpretation. Trajectory-based and zone-based event reasoning have been widely used in human–object interaction analysis, traffic monitoring, people counting, and surveillance. Bernardin and Stiefelhagen [36] developed the CLEAR MOT measurements, which helped standardize tracking system evaluation and illustrated the need for event-level interpretation in multi-object situations. While Milan et al. [37] presented the MOT16 benchmark for multi-object tracking, Dendorfer et al. [38] suggested MOT20 for congested tracking situations. These criteria are helpful for evaluating identity retention and localization, but they do not address domain-specific event interpretation in retail billing.

The interpretability of zone-based reasoning makes it attractive in smart-shelf and retail environments. The symbolic sequence that arises from mapping a product track to locations like shelf, middle, and exit can be interpreted using finite-state principles. Unlike black-box video classifiers, this type of reasoning allows for the examination of every decision. For example, a shift from shelf to middle to exit can be classified as a product removal, while a shift from shelf to middle and back to shelf can be classified as a returned product. Multi-camera fusion is another important field of research. Multi-camera systems have extra challenges with camera calibration, synchronization, and cross-view association, even though they can reduce viewpoint ambiguity and occlusion. Stereo reconstruction is challenging in small smart vending cabinets due to the limited baseline, potentially asynchronous cameras, and hand occlusion, condensation, and reflections that deteriorate visual correspondences. As a result, event-level fusion may be more useful than 3D geometric fusion or pixel-level fusion.

The proposed ZAB-Fusion framework is at the center of these investigations. Although it uses ByteTrack for temporal association, it does not stop at trajectory generation. A finite-state event interpreter is utilized to classify the output, each product track is mapped into a three-zone cabinet model, the zone sequence is compressed, and the two camera-specific event streams are combined using evidence-gated cross-camera fusion. The outcome is an auditable receipt-level decision instead of a collection of trajectories, which sets it apart from conventional multi-object tracking methods.

Unlike previous product identification datasets and object detection algorithms, ZAB-Fusion prioritizes decision-level reliability. Unlike general multi-object tracking methods, it introduces domain-specific event semantics. Unlike learning multi-camera fusion, it uses an interpretable rule-based event-fusion technique suitable for commercial invoicing. Therefore, this work’s primary contribution is a transaction-level reasoning framework for sensorless smart vending networks rather than a novel detector or tracker.

## 3. System Architecture and Problem Formulation

The system-level architecture of the suggested smart vending transaction recognition framework is explained in this section. The top-loading refrigerated vending cabinet’s hardware configuration is first described. Next, a formal definition of the video-based transaction recognition problem is given. Lastly, the constraints of the phantom-VETO failure mode and the snapshot-difference baseline are described.

### 3.1. Smart Vending Cabinet Hardware Configuration

A top-loading refrigerated smart vending cabinet intended for unattended retail operation is the target platform. Using a QR code, NFC card, or smartphone app, a consumer unlocks the cabinet, opens the lid, interacts with the merchandise, and then shuts the lid to complete the transaction. The system must identify the products that were taken and produce the final product-level receipt once the lid is closed. The cabinet is purposefully sensorless from the standpoint of product recognition. Weight sensors, shelf load cells, product-slot instrumentation, and item-level RFID tags are not used. As a result, all transaction decisions must be made based on visual data that the cameras inside the cabinet have recorded. Because more sensors raise hardware costs, installation complexity, maintenance effort, and calibration needs, this limitation is crucial for practical implementation. Two cameras are installed beneath the lid of the cabinet. While Camera 1 is positioned with an approximately 45°convergent oblique perspective, Camera 0 offers a nearly perpendicular top-down view of the product shelf. The two cameras are separated by a horizontal baseline of 235 mm. Both cameras record at about 29 frames per second with a native resolution of 1280 × 720 pixels. Nevertheless, there is no presumption of frame-level synchronization because the two video streams are asynchronous. This geometry motivates event-level fusion instead of stereo-level fusion by defining Camera 0 as a perpendicular view and Camera 1 as a 45° convergent view with a 235 mm baseline.

**Figure 1:**
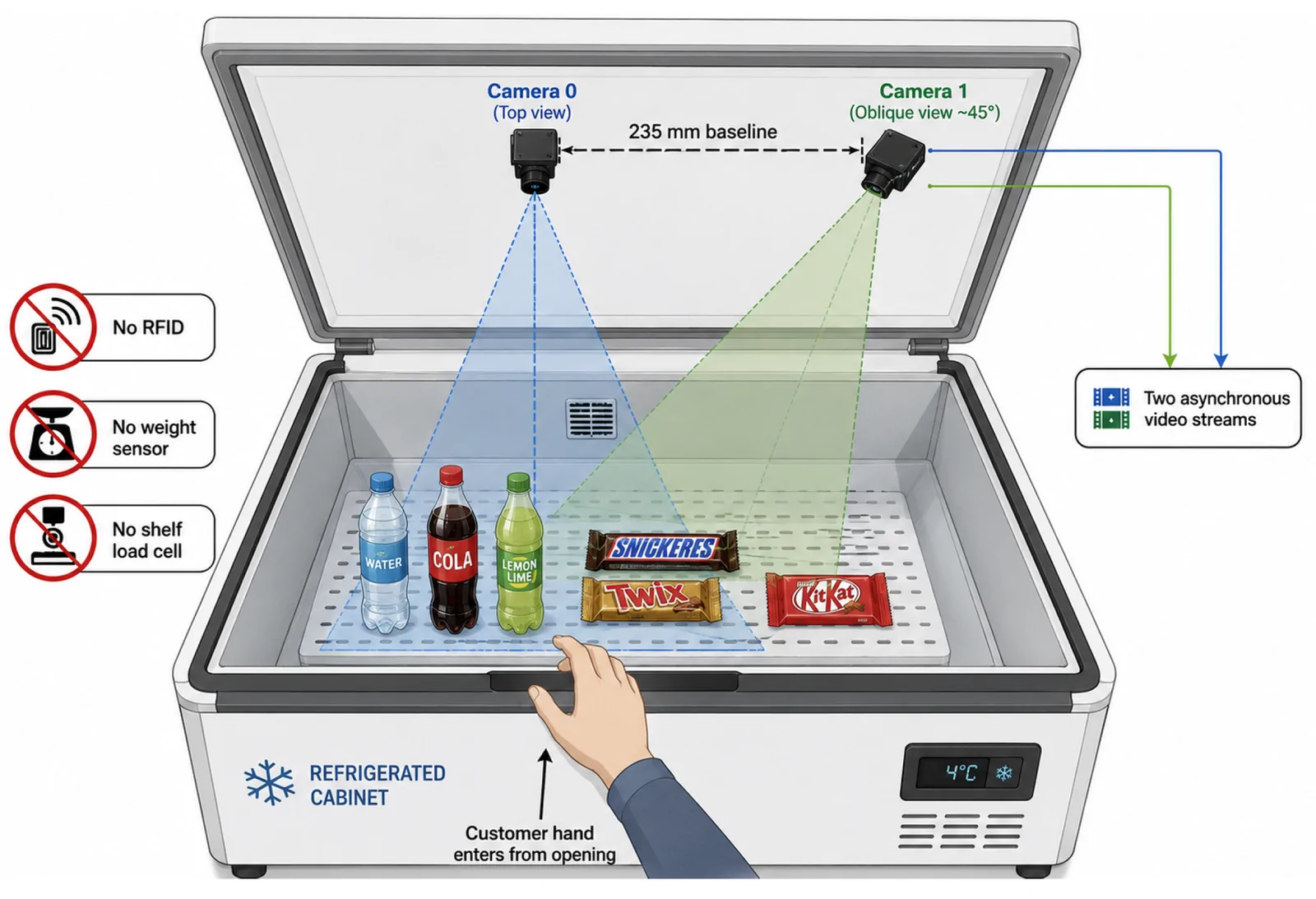
Hardware configuration of the top-loading smart vending cabinet.

Figure 1 shows the cabinet’s hardware arrangement. The two cameras are regarded as separate visual observers of the same transaction. The system does not estimate depth using disparity because reliable stereo reconstruction is challenging due to the limited baseline, convergent geometry, hand occlusions, reflections, and condensation. Rather, each video stream is analyzed separately, and at the event-decision level, the outcomes are then combined.

This geometry motivates event-level fusion rather than stereo-level fusion because Camera 0 provides a near-perpendicular view, Camera 1 provides an approximately 45° convergent view, and the two asynchronous streams do not provide reliable conditions for disparity-based depth estimation. There is a 235 mm baseline between the two cameras. All transaction decisions must be deduced from the two asynchronous video streams since the system does not use RFID tags, weight sensors, shelf load cells, or product-slot instrumentation. To create a uniform coordinate standard, every video frame is 180° rotated before processing. The departure direction appears at the bottom of the rotated frame, whereas the shelf region looks close to the top. Y is a vertical coordinate that rises downward. Zone-based motion interpretation subsequently makes use of this coordinate standard.

### 3.2. Video-Based Transaction Recognition Problem

The goal of the system is to infer the set of products taken by the customer during a transaction. Let the two asynchronous video streams be denoted by V_0_ = {f_0_^1^, f_0_^2^, …, f_0_^N^} and V_1_ = {f_1_^1^, f_1_^2^, …, f_1_^M^}, where V_0_ and V_1_ are the video streams captured by Camera 0 and Camera 1, respectively; f_i_^t^ denotes the t-th frame from camera i; and N and M are the numbers of frames in the two streams. Since the cameras are asynchronous, N and M may differ, and a direct correspondence between f_0_^t^ and f_1_^t^ is not required. Let the product catalogue be represented by C = {c_1_, c_2_, …, c_K_}, where each c_k_ denotes one product class. In the evaluated cabinet, the catalogue contains seven product classes: 18 + 0.45 L, Chortoq 0.5 L, Fanta 0.5 L, Moxito 0.5 L, Snickers 80 g, Super Kontik 100 g, and Twix 75 g. The desired output is a transaction-level receipt: R = {(c, q_c_)|c ∈ C, q_c_ ∈ ℕ_0_}, where q_c_ is the estimated quantity of product class c taken by the customer. A product must be counted if it is removed from the cabinet. However, it must not be counted if it is only inspected, partially lifted, shifted on the shelf, temporarily occluded, or returned before the transaction ends.

**Figure 2:**
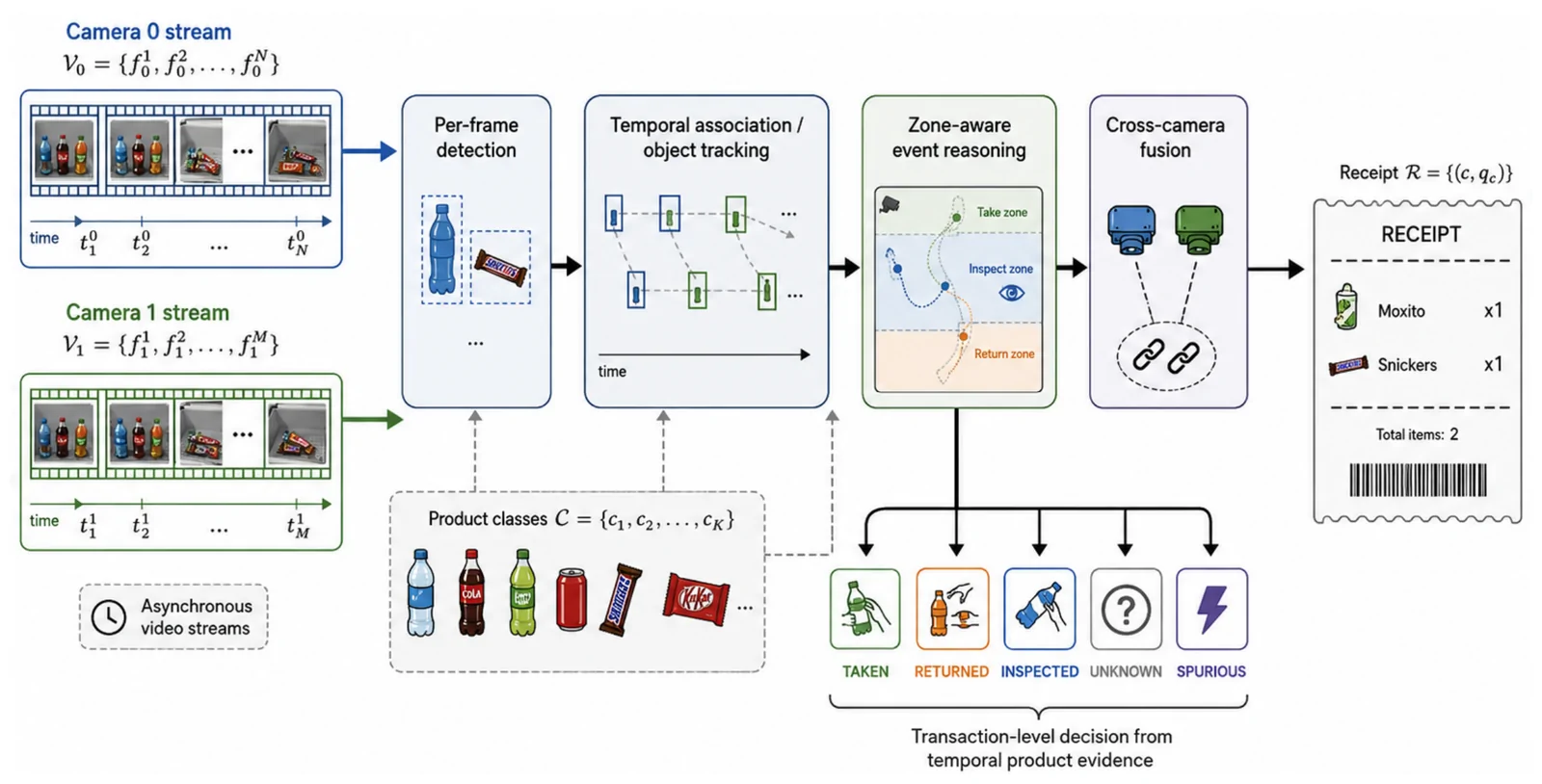
Video-based transaction recognition problem formulation.

The overall input-output formulation is shown in Figure 2. The key point is that the final receipt is not a direct object detection result. It is a transaction-level decision derived from temporal product evidence.

The system receives two asynchronous video streams, V_0_ and V_1_, from Camera 0 and Camera 1. For each stream, frame-level product detections are associated over time to form object tracks. These tracks are then converted into semantic product events such as TAKEN, RETURNED, INSPECTED, UNKNOWN, or SPURIOUS. The final output is a product-level receipt R = {c, q_c_}, which contains the estimated product class and quantity taken by the customer. This formulation demonstrates that the task is not merely an object detection problem. A detector can identify products in individual frames, but it cannot directly determine whether a detected product was taken, returned, inspected, or only temporarily moved. Therefore, transaction recognition requires temporal association and semantic interpretation of product trajectories. For this reason, the event space is defined as E = {TAKEN, RETURNED, INSPECTED, UNKNOWN, SPURIOUS}. Here, TAKEN indicates that the product was removed from the cabinet; RETURNED indicates that the product was lifted and placed back on the shelf; INSPECTED indicates temporary interaction without removal; UNKNOWN indicates incomplete or ambiguous evidence; and SPURIOUS indicates a noise track or false trajectory.

The complete transaction recognition problem can therefore be written as F: (V_0_, V_1_) → R, where F consists of frame-level detection, temporal association, zone-aware trajectory interpretation, event classification, and evidence-gated cross-camera fusion.

### 3.3. Snapshot-Difference Baseline

A common production-level baseline for smart vending recognition is the snapshot-difference approach. In this method, the system samples one or several frames from the beginning of the transaction and one or several frames from the end. A product detector is applied to these frames, and the difference between the detected initial and final product sets is interpreted as the customer’s basket. Let S_start_ denote the detected product multiset at the beginning of the transaction and let S_end_ denote the detected product multiset at the end. The snapshot-difference receipt can be expressed as R_diff_ = S_start_ − S_end_, where the multiset difference represents products inferred to have been removed from the cabinet.

The main advantage of this strategy is simplicity. It requires only frame-level object detection and does not require tracking, trajectory modelling, zone reasoning, or evidence-gated cross-camera fusion. As a result, it is computationally attractive for low-power edge systems. However, the method has a structural limitation: it ignores the entire temporal interval between the initial and final observation windows. Therefore, it cannot distinguish between visually similar endpoint states produced by different customer actions. For example, the following actions may produce the same or nearly the same initial and final shelf appearance:

- A product is picked up and taken;
- A product is picked up, inspected, and returned;
- A product is partially lifted and placed back;
- A neighboring product shifts after a pickup;
- A small item is removed but not detected due to occlusion or scale;
- The customer’s hand temporarily hides a product during the final frame.

Since these intermediate events are not represented in S_start_ or S_end_, the snapshot-difference method cannot recover them at the decision stage. This means that the failure is not only a detector-level error but also a decision-level error caused by the absence of motion semantics.

In this study, snapshot-difference refers to the endpoint-only baseline that compares detected product sets at the beginning and end of a transaction without modeling intermediate motion. The term phantom-VETO refers to failure cases in which the initial and final shelf states appear visually acceptable, but the intermediate product trajectory contains essential transaction evidence that is ignored or suppressed by endpoint-only reasoning. After these definitions, the terms snapshot-difference and phantom-VETO are used consistently throughout the manuscript. The snapshot-difference baseline compares only the initial and final observation windows of a transaction. Product interactions occurring between these windows are ignored. As a result, pickup–return, inspection, temporary occlusion, shelf re-settling, or small-item removal events may be missed or incorrectly suppressed. This endpoint-only limitation is referred to as the phantom-VETO problem.

**Figure 3:**
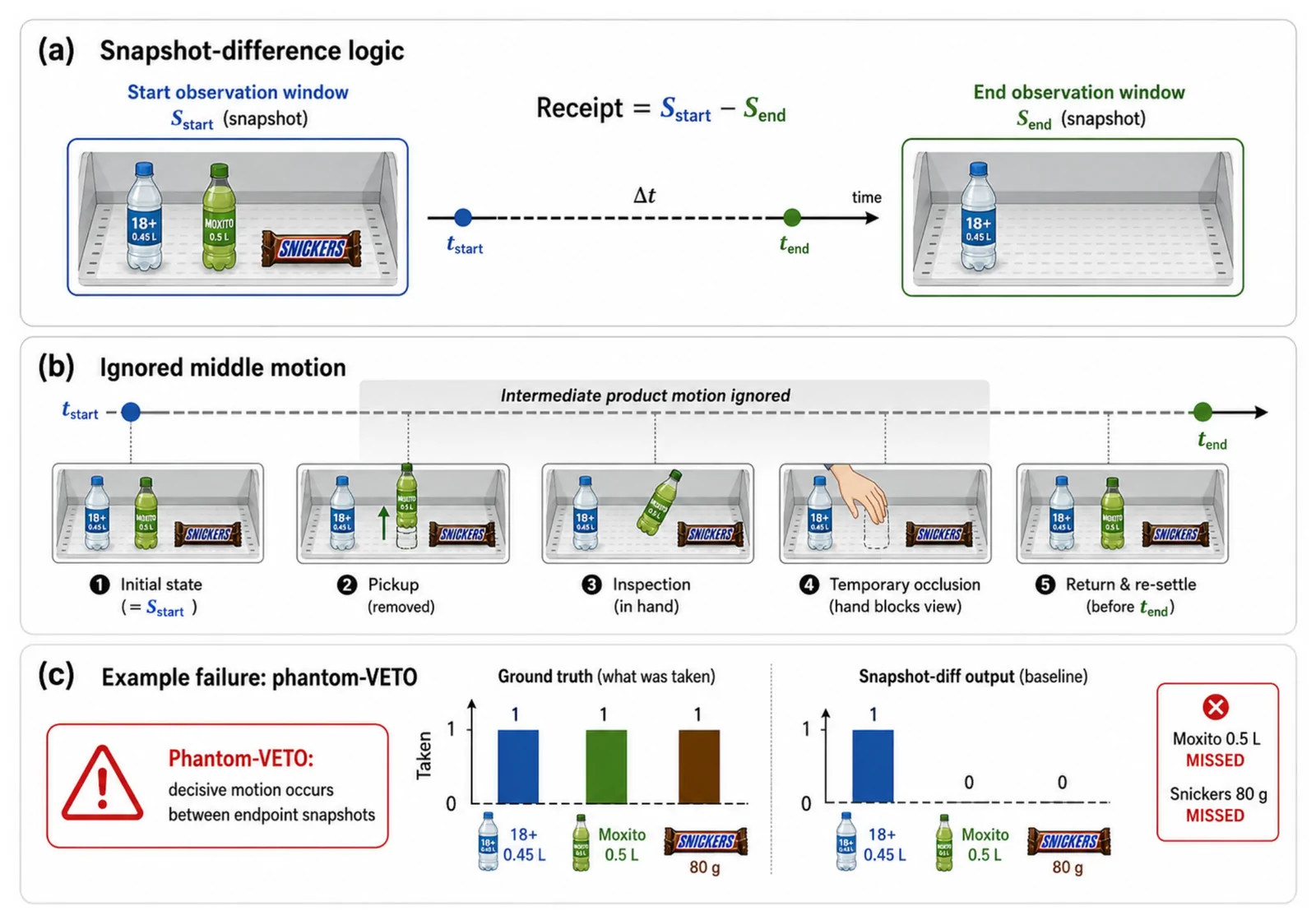
Snapshot-difference baseline and phantom-VETO failure mode: (**a**) Snapshot-difference logic, (**b**) Ignored middle motion, (**c**) Failure-phantom VETO.

The phantom-VETO problem is particularly important in top-loading smart vending cabinets for several reasons. First, customer interactions are short and frequently involve hand occlusion. A product may be visible only for a few frames while it is being removed from the shelf. If this motion does not appear clearly in the endpoint snapshots, the baseline cannot identify the pickup. Second, small products such as confectionery items occupy only a small region in the image. In the studied cabinet, small items may appear only approximately 25–40 pixels high in some views, making them difficult to detect reliably, especially under partial occlusion. Third, products may be inspected or returned. A customer may lift a product, move it toward the exit region, and then place it back on the shelf. In this case, the initial and final shelf states may appear similar, but the intermediate trajectory contains meaningful interaction evidence. Fourth, products may shift without being purchased. When the customer removes one item, neighboring products may move or settle into a new position. A snapshot-based system may incorrectly interpret such re-settling as a purchase or may suppress a true pickup because the final shelf configuration still appears visually plausible. The real in-service transaction WZ1776169039764 demonstrates this failure mode. In this transaction, the snapshot-difference baseline detected only one visually distinctive product, while two other ground-truth pickups, Moxito 0.5 L and Snickers 80 g, were missing. This example confirms that endpoint-only reasoning is insufficient for dense, occluded, and motion-rich vending scenes (Figure 3).

### 3.4. Design Requirements for the Proposed Framework

Based on the limitations of the snapshot-difference baseline, the proposed transaction recognition framework must satisfy six design requirements. First, it must use continuous temporal evidence instead of relying only on the beginning and ending frames. Product motion during the transaction must be explicitly represented. Second, it must convert low-level detections into object-level trajectories. Since the billing decision concerns product instances rather than isolated bounding boxes, frame-level detections must be associated over time. Third, it must interpret trajectories using motion semantics. The system must distinguish between products that are taken, returned, inspected, ambiguous, or spurious. Fourth, it must handle the asymmetric geometry of the two-camera setup. Camera 0 and Camera 1 observe the same shelf from different angles, so a single geometric threshold may not be appropriate for both views. Fifth, it must perform auditable cross-camera fusion. Since billing decisions may be disputed by customers, the final receipt should be explainable in terms of camera-specific tracks, zone transitions, and event labels. Sixth, it must remain suitable for CPU-only edge deployment. The additional reasoning stages should not impose a significant computational burden beyond the detector ensemble.

These requirements motivate the proposed ZAB-Fusion framework, which is described in the next section. ZAB-Fusion combines frame-level detection, multi-object tracking, zone-aware trajectory representation, finite-state event classification, and evidence-gated cross-camera fusion to infer the final product-level receipt from two asynchronous video streams.

## 4. Proposed ZAB-Fusion Framework

**Figure 4:**
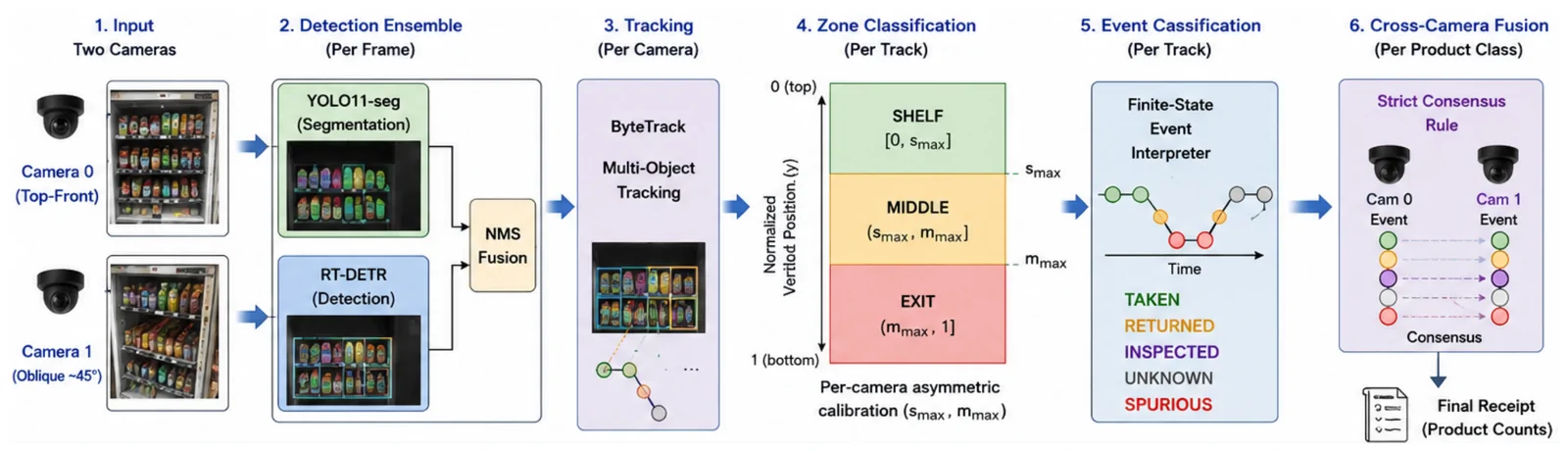
Overall architecture of the proposed ZAB-Fusion framework.

This section presents the proposed ZAB-Fusion framework for sensorless smart vending transaction recognition. The framework converts two asynchronous video streams into an auditable product-level receipt through frame-level detection, temporal association, zone-aware track representation, finite-state event classification, and evidence-gated cross-camera fusion. Unlike snapshot-difference methods, ZAB-Fusion uses continuous temporal evidence and explicitly models product motion during the transaction. The complete processing flow of the proposed framework is shown in Figure 4. Each camera stream is processed independently until the event level. The resulting camera-specific event streams are then merged by the evidence-gated cross-camera fusion module.

Each asynchronous camera stream is processed through per-frame detection, ByteTrack-based temporal association, zone-aware motion representation, and finite-state event classification. Camera-specific events are then merged using evidence-gated cross-camera fusion to generate the final transaction receipt.

### 4.1. Per-Frame Detection Ensemble

The first stage of ZAB-Fusion is frame-level product detection. Each selected and rotated video frame is processed using two complementary object detectors: YOLO11-seg and RT-DETR. YOLO11-seg provides stable localization for larger bottle-like products, whereas RT-DETR provides complementary detection evidence for small or partially occluded confectionery products. The detector ensemble is used because the evaluated product catalogue contains both large beverage bottles and small packaged items, which differ substantially in scale, shape, texture, and visual saliency.

Let $f_{t}$ denote a selected video frame at time *t.* The detector outputs are represented as sets of bounding boxes, class labels, and confidence scores:

$$D_{t}^{Y}\;=\left\{\left(b_{i}\;,c_{i}\;,p_{i}\;\right)\right\},$$

$$D_{t}^{R}=\left\{\left(b_{j}\;,\;c_{j}\;,p_{j}\;\right)\right\},$$

where *b* is the bounding box, *c* is the predicted product class, and *p* is the detection confidence. Before merging the detector outputs, detections with confidence lower than 0.35 were removed. The same confidence threshold was used for both YOLO11-seg and RT-DETR and for all seven product classes; no detector-specific or class-specific confidence threshold was used. The value 0.35 was selected using the validation subset. Lower thresholds increased the number of candidate detections for small products but also introduced more short false tracks caused by reflections, shelf re-settling, and hand-induced motion. Higher thresholds suppressed false detections but reduced recall for small confectionery products and partially occluded items. Therefore, 0.35 was chosen as a validation-based operating point that balances small-product recall and billing-oriented false-positive control. After confidence filtering, the YOLO11-seg and RT-DETR outputs were merged using class-aware non-maximum suppression with an IoU threshold of 0.45:

$$D_{t}={NMS}_{IoU=0.45}\;(D_{t}^{Y}\;\cup D_{t}^{R}\;).$$

The resulting detection set $D_{t}\;$ was then passed to ByteTrack for temporal association. The detection ensemble itself is not the main novelty of this work. Instead, it provides the frame-level product evidence used by the subsequent tracking, zone-state interpretation, and evidence-gated cross-camera fusion stages. The key contribution of ZAB-Fusion is how these detections are transformed into transaction-level product events.

### 4.2. ByteTrack-Based Temporal Association

After per-frame detection, ZAB-Fusion applies ByteTrack to associate detections across frames and form object trajectories. This step is necessary because billing decisions concern product instances, not isolated frame-level detections. For a complete transaction, ByteTrack produces a set of product tracks:

*T* = {*τ*_1_, *τ*_2_, …, *τL*},

where each track *τ_k_* is an ordered sequence of observations:

*τ_k_* = [(*t_j_*, *b_j_*, *c_j_*, *p_j_*)], *j* = 1…*n_k_*.

Here, *t_j_* is the frame index, *b_j_* is the bounding box, *c_j_* is the detected product class, *p_j_* is the confidence score, and *n_k_* is the length of the track. The class label of a track is assigned by majority voting over its frame-level detections:

*cls*(*τ_k_*) = *mode* (*c*_1_, *c*_2_, …, *c_nk_*).

The mean confidence of the track is computed as

*Conf*^−^(*τ_k_*) = (1/*n_k_*) Σ*j* = 1…*n_k_ p_j_.*

Tracks shorter than five frames or with mean confidence below 0.35 are treated as unreliable candidates and later classified as SPURIOUS. This filtering suppresses short ghost tracks produced by detector noise, reflections, or momentary occlusion.

ByteTrack is suitable for the vending cabinet scenario because it can associate both high-confidence and low-confidence detections. This is important when a customer’s hand partially includes products for several frames. Instead of immediately terminating a track, ByteTrack can preserve identity through short-term uncertainty, allowing the downstream event classifier to interpret the full trajectory.

### 4.3. Zone-Aware Track Representation

The central idea of ZAB-Fusion is to represent product motion using symbolic cabinet zones. Since the task is to determine whether a product moves from the shelf toward the exit, a full pixel-level trajectory is not required. Instead, each track observation is mapped to one of three vertical zones: SHELF, MIDDLE, and EXIT. The SHELF zone corresponds to the product storage region. The MIDDLE zone corresponds to the intermediate movement region where products are typically lifted, inspected, or carried by the customer’s hand. The EXIT zone corresponds to the region through which a product leaves the cabinet. For each bounding box observation, the bottom-edge coordinate is used because it better represents the physical support/contact position of the product in the cabinet image. Let y_2_ be the bottom coordinate of a bounding box and H be the image height. The normalized vertical coordinate is

*y_norm_* = *y*_2_/*H*.

The zone assignment function is defined as

*Z*(*y_norm_*) = *SHELF*, *if y_norm_* < *s_max_*,

*Z*(*y_norm_)* = *MIDDLE*, *if s_max_* ≤ *y_norm_* < *m_max_*,

*Z*(*y_norm_*) = *EXIT*, *if y_norm_* ≥ *m_max_*.

The parameters *s_max_* and *m_max_* define the vertical zone boundaries. In the released configuration, the zone boundaries are camera-specific:

*Camera* 0: *s_max_* = 0.62, *m_max_* = 0.82;

*Camera* 1: *s_max_* = 0.65, *m_max_* = 0.82.

The asymmetric value of *s_max_* is necessary because Camera 0 and Camera 1 observe the shelf from different angles. Camera 0 provides a near-perpendicular view, while Camera 1 observes the scene from an approximately 45° convergent angle. Therefore, the same physical shelf area occupies different vertical proportions in the two image planes. Applying the same zone boundary to both cameras would incorrectly place some static shelf products into the MIDDLE zone, producing false motion events.

**Figure 5:**
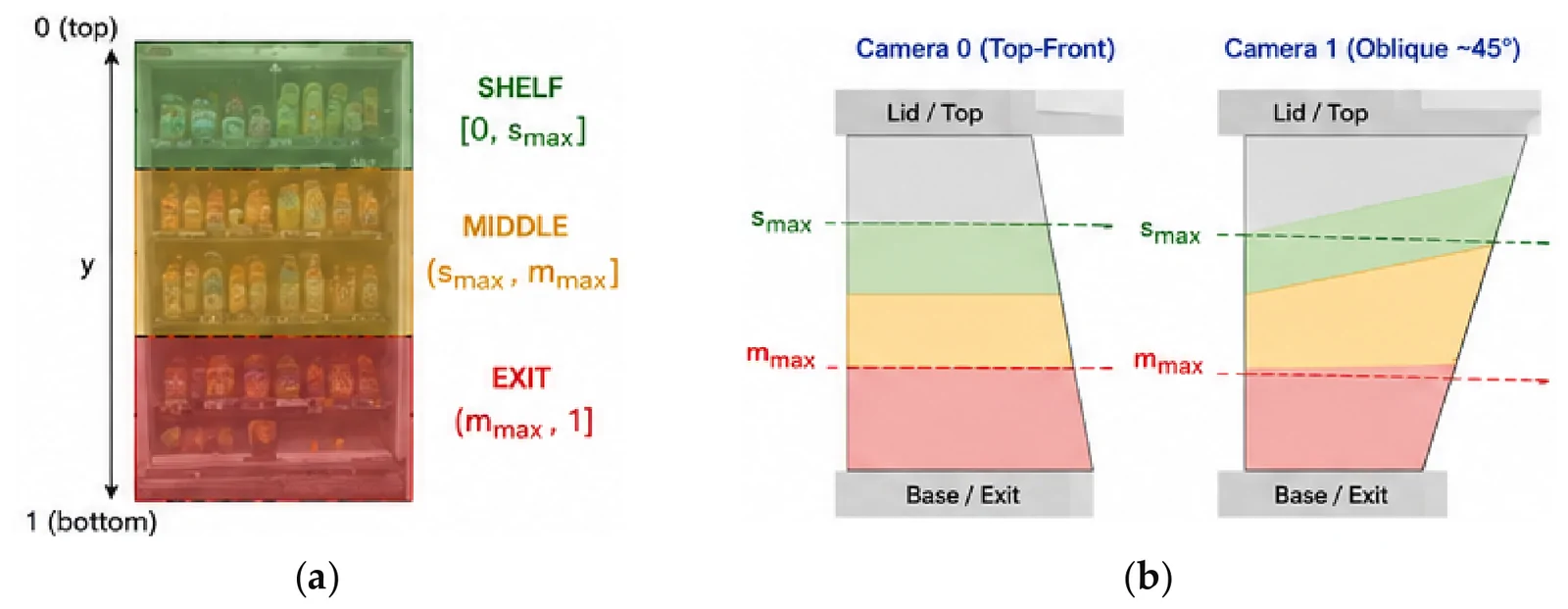
Zone-aware track representation: (**a**) vertical zones in normalized image; (**b**) different viewpoint.

The zone-aware representation is shown in Figure 5.

Each product track is mapped into one of three vertical cabinet zones using the normalized bottom-edge coordinate of the bounding box. Repeated zone labels are compressed into a symbolic sequence, which is later interpreted by the finite-state event classifier. For a track *τ_k_*, the raw zone sequence is *Z*(*τ_k_*) = [*z*_1_, *z*_2_, …, *z_nk_*], where *z_j_* ∈ {*SHELF*, *MIDDLE*, *EXIT*}. Consecutive repeated zones are removed to obtain a compressed symbolic sequence: *Z*(*τ_k_*) = *compress*(*Z*(*τ_k_*)). For example,

[*SHELF*, *SHELF*, *MIDDLE*, *MIDDLE*, *EXIT*, *EXIT*] → [*SHELF*, *MIDDLE*, *EXIT*].

The compressed sequence preserves the semantic structure of the trajectory while removing redundant frame-level repetitions.

### 4.4. Finite-State Event Classification

The finite-state event classifier converts each compressed zone sequence into a semantic product event. The classifier is rule-based and fully interpretable. This is important because vending transactions may lead to customer disputes, and each billing decision must be explainable. The event space is defined as

E = {TAKEN, RETURNED, INSPECTED, UNKNOWN, SPURIOUS}.

The mapping between compressed zone sequences and event labels is summarized in Table 1.

A track is classified as TAKEN when its movement indicates removal from the shelf toward the exit. A track is classified as RETURNED when the object leaves the shelf but returns to it. A track is classified as INSPECTED when it moves toward the exit direction but is ultimately placed back. An UNKNOWN event preserves weak evidence that cannot be confidently interpreted by a single camera. A SPURIOUS event suppresses static objects, ghost tracks, and unreliable detections.

The rule-based classifier can be written as

*e_k_* = *FSM*(*Z*^−^(*τ_k_*), *len*(*τ_k_*), *conf*^−^(*τ_k_*)),

where *e_k−_* is the event label assigned to track *τ_k_*.

The output of this stage for each camera is a camera-specific event stream:

*E_i_* = {(*e_k_*, *cls*(*τ_k_*), *id*(*τ_k_*), *conf*^−^(*τ_k_*))},

where *i* ∈ {0, 1} denotes the camera index.

### 4.5. Evidence-Gated Cross-Camera Fusion

After the two camera streams are processed independently, ZAB-Fusion obtains two event streams: *E*_0_ from Camera 0 and *E*_1_ from Camera 1. Since the two cameras are asynchronous and the narrow-baseline cabinet geometry is not suitable for reliable stereo reconstruction, the proposed method performs fusion at the symbolic event level rather than at the pixel, feature, or 3D geometry level.

We use the term evidence-gated cross-camera fusion because the rule is conservative but not a pure intersection rule. It allows strong single-camera evidence under controlled conditions, while requiring confirmation for weak or ambiguous evidence. This terminology better reflects the intended billing-oriented behavior of the fusion stage.

Let *e_i_*,*k* = (*c_i_*,*k*, *l_i_*,*k*, *s_i_*,*k*, *z_i_*,*k*) denote the *k-th* event generated by camera i, where i ∈ {0,1}. Here, c_i_,k is the predicted product class, *l_i_*,*k* ∈ {*TAKEN*, *RETURNED*, *INSPECTED*, *UNKNOWN*, *SPURIOUS*} is the event label, *s_i_*,*k* is the mean track confidence, and *z_i_*,*k* is the compressed zone sequence associated with the event. The purpose of the fusion module is to convert the two event streams *E*_0_ and *E*_1_ into the final transaction receipt R.

The proposed fusion rule distinguishes four evidence cases.

Case 1: Confirmed two-camera TAKEN evidence. If both cameras independently produce a TAKEN event for the same product class c within the same transaction, the event is accepted as confirmed two-camera evidence:

$$TAKEN_{0}\left(c\right)\wedge \;TAKEN_{1}\left(c\right)\to \;ACCEPT\left(c\right).$$

This case provides the strongest evidence because the same product-removal event is supported by both views.

Case 2: Admitted single-camera TAKEN evidence. If one camera produces a high-confidence TAKEN event for product class c and the opposite camera provides no contradictory evidence for the same class, the event is accepted as admitted single-camera evidence:

$$TAKEN_{i}\left(c\right)\wedge \;\neg CONTRADICT_{1-i}\left(c\right)\to \;ACCEPT\left(c\right),$$

where *i* ∈ {0, 1}. A contradictory event is defined as an opposite-camera RETURNED or INSPECTED event for the same product class that clearly indicates non-removal. This admitted single-camera case is necessary because one camera may be temporarily occluded by the customer’s hand, affected by reflection, or unable to observe the product trajectory clearly.

Case 3: UNKNOWN-supported evidence. An UNKNOWN event is treated as weak evidence and is not accepted by itself. It is accepted only when the opposite camera independently produces a TAKEN event for the same product class:

$$UNKNOWN_{i}\left(c\right)\wedge \;TAKEN_{1-i}\left(c\right)\to \;ACCEPT\left(c\right).$$

This rule allows incomplete single-view evidence to support a product-removal decision only when the other view provides strong evidence.

Case 4: Rejected ambiguous evidence. Isolated UNKNOWN events, isolated SPURIOUS events, static shelf tracks, and contradictory events without TAKEN support are rejected:

$$UNKNOWN_{i}(c)\;\wedge \;\neg TAKEN_{1-i}(c)\;\to \;REJECT(c),$$

$$SPURIOUS_{i}(c)\;\to \;REJECT(c).$$

This prevents weak single-camera evidence from being directly converted into a billed item. The final quantity $q_{c}$ for each product class c is computed from the number of accepted TAKEN-level decisions:

$$q_{c}=\;N_{accept\left(c\right)},$$

where $N_{accept\left(c\right)},$ denotes the number of accepted product-removal events of class c after evidence-gated fusion. The final receipt is then defined as

$$R=\left\{\left(c,\;q_{c}\right)\right|c\in C,\;q_{c}>0\},$$

where *c* is the product class and $q_{c}$ is the predicted quantity of class *c* removed from the cabinet.

This fusion rule is intentionally different from both symmetric union and strict intersection. A symmetric union rule accepts all candidate events from either camera and may increase false positives, which is undesirable in a billing system. A strict intersection rule accepts only events confirmed by both cameras and may miss genuine pickups when one camera is occluded or temporarily unreliable. The proposed evidence-gated fusion rule provides an intermediate operating point: strong TAKEN evidence can be admitted, weak UNKNOWN evidence requires confirmation, and ambiguous or contradictory evidence is rejected.

**Figure 6:**
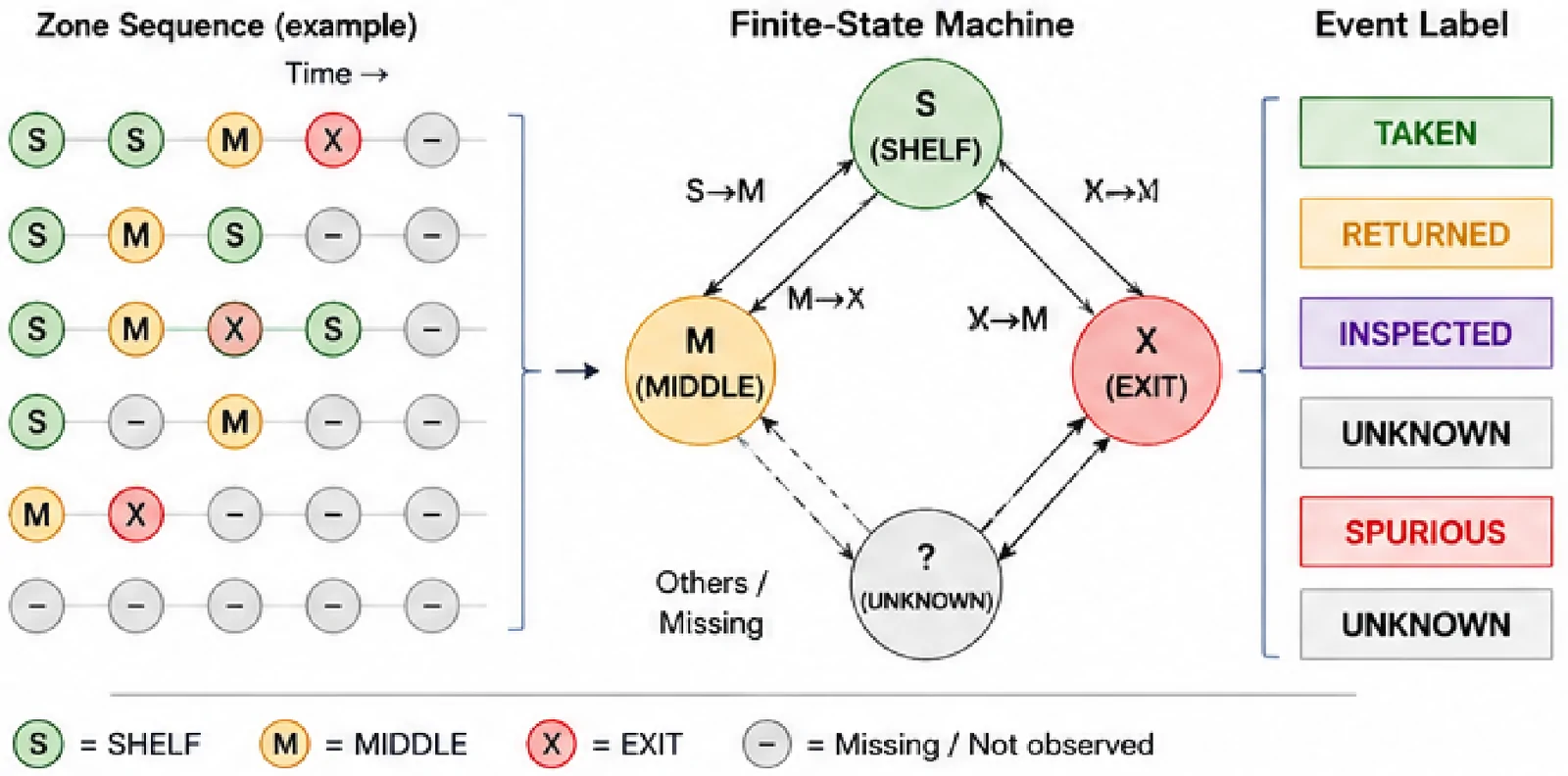
Evidence-gated cross-camera fusion rule.

This design also improves auditability. Each accepted receipt item can be traced back to the camera source, track identifier, product class, compressed zone sequence, event label, confidence score, and fusion decision. Therefore, the final billing output is not only a product count but also an interpretable decision supported by camera-specific motion evidence. The evidence-gated cross-camera fusion rule is shown conceptually in Figure 6.

The fusion rule accepts confirmed two-camera TAKEN evidence, admits high-confidence single-camera TAKEN evidence when no contradictory evidence exists, accepts UNKNOWN evidence only with opposite-camera TAKEN confirmation, and rejects isolated weak or spurious events. The complete ZAB-Fusion procedure is summarized in Algorithm 1.

**Algorithm 1.** ZAB-Fusion transaction recognition.Input: Two asynchronous video streams V_0_, V_1_; product catalogue C; camera-specific zone parameters (s_max_, m_max_). Output: Final transaction receipt R.1.  For each camera stream V_i_, where i ∈ {0,1}:2.  For each frame f_i_^t^ in V_i_:3.  Rotate the frame by 180° to establish the common coordinate convention.4.  Apply YOLO11-seg to obtain product detections.5.  Apply RT-DETR to obtain product detections.6.  Merge detector outputs using NMS to obtain D_i_^t^.7.  Apply ByteTrack to associate detections over time and generate tracks T_i_.8.  For each track τ_k_ ∈ T_i_:9.  Compute track length len(τ_k_) and mean confidence conf^−^(τ_k_).10.   If len(τ_k_) < 5 or conf^−^(τ_k_) < 0.35, assign SPURIOUS.11.   Otherwise, compute y_norm = y_2_/H for each track observation.12.   Map each observation to SHELF, MIDDLE, or EXIT using the camera-specific zone boundaries.13.   Compress repeated zone labels to obtain Z^−^(τ_k_).14.   Apply the finite-state classifier to assign an event label e_k_.15.   Store the camera-specific event (e_k_, cls(τ_k_), id(τ_k_), conf^−^(τ_k_)).16.   After both cameras are processed, obtain event streams E_0_ and E_1_.17.   Admit all valid TAKEN events.18.   Admit UNKNOWN events only if the opposite camera confirms a TAKEN event of the same class.19.   Compute the final quantity q_c for each product class using the evidence-gated cross-camera count rule.20.   Return R = {(c, q_c_)|q_c_ > 0}.

The current evidence-gated fusion rule generates the final receipt by counting accepted TAKEN-level events for each product class. Each accepted event is associated with its camera source, track identifier, product class, compressed zone sequence, event label, confidence score, and fusion decision. This design provides an auditable product-level receipt and works well when product trajectories are sufficiently separated. However, the current implementation does not fully solve the problem of distinguishing multiple identical units when several products of the same SKU are removed consecutively or simultaneously under severe occlusion. For example, if two visually identical items are picked up in close temporal proximity and their tracks overlap or fragment, the system may incorrectly merge two physical product removals into one accepted class-level event, or may split one physical removal into multiple candidate events. This limitation is particularly relevant for small confectionery products and densely arranged shelf layouts. Therefore, the present fusion rule should be understood as an event-level and class-level billing mechanism rather than a complete multi-instance identity-resolution method for all identical-product scenarios. To improve robustness in such cases, future versions of the framework should incorporate trajectory-level instance separation, cross-view track matching, temporal spacing constraints, and product-instance re-identification. These extensions would allow the system to better distinguish independent trajectories of multiple identical products and reduce counting errors in consecutive same-SKU pickup scenarios.

## 5. Experimental Setup

This section describes the experimental setup used to evaluate the proposed ZAB-Fusion framework. The evaluation is based on real in-service transactions collected from a deployed smart vending cabinet. The section presents the transaction dataset, product catalogue, annotation protocol, baseline configurations, evaluation metrics, and implementation environment.

### 5.1. In-Service Transaction Dataset

The evaluation dataset consists of 220 complete in-service customer transactions collected from a deployed top-loading smart vending cabinet (Table 2). Each transaction corresponds to the full interaction interval between lid opening and lid closing. For every transaction, two videos were recorded simultaneously by Camera 0 and Camera 1. Both cameras captured frames at a native resolution of 1280 × 720 pixels and approximately 29 frames per second. The two camera streams were treated as asynchronous and were not frame-synchronized during processing. The dataset contains 227 ground-truth TAKEN events and 87 RETURNED events, corresponding to 314 annotated transaction-level events across seven product classes. The product catalogue consists of 18 + 0.45 L, Chortoq 0.5 L, Fanta 0.5 L, Moxito 0.5 L, Snickers 80 g, Super Kontik 100 g, and Twix 75 g. The collected transactions include clean single-product pickups, multiple-item pickups, pickup-and-return sequences, inspection actions, temporary hand occlusions, and shelf re-settling events. These cases are important because they represent the types of motion ambiguity that make endpoint-only snapshot-difference recognition unreliable. After removing lid-opening and lid-closing buffer frames, the average transaction duration was 27.4 s, corresponding to approximately 800 frames per camera per transaction. This duration refers to the recorded transaction video length, not the processing latency. The processing latency of the proposed system is reported separately in the runtime analysis.

Although the dataset contains 220 in-service transactions and 314 annotated transaction-level events, the evaluation is still limited to one cabinet and one deployment site. Therefore, the results should be interpreted as single-site in-service validation of the proposed dual-camera edge sensing framework. Therefore, the results should be interpreted as a single-site in-service validation of the proposed dual-camera edge sensing framework. Multi-cabinet, multi-site, and multi-condition validation remains an important direction for future work.

The smart vending cabinet used in this study contains seven product classes. The catalogue includes both large and small objects, which makes the recognition problem challenging. Large bottle-like products are easier to localize because they occupy a larger image region and have clearer vertical structure. In contrast, small confectionery items occupy only a limited number of pixels, especially in the oblique camera view, and are more sensitive to occlusion, blur, and packaging similarity.

The product catalogue is defined as *C* = {*c*_1_, *c*_2_, …, *c*_7_}, where the seven classes are

1. 18 + 0.45 L;
2. Chortoq 0.5 L;
3. Fanta 0.5 L;
4. Moxito 0.5 L;
5. Snickers 80 g;
6. Super Kontik 100 g;
7. Twix 75 g.

The inclusion of small confectionery products is important for evaluating the proposed framework because snapshot-difference methods frequently miss small items when they are occluded or located in lower shelf rows.

To ensure reproducibility, the detector-training, inference, and tracking settings are explicitly reported. The detection stage used YOLO11s-seg and RT-DETR-R18 as complementary product detectors. Both models were initialized from their publicly available COCO-pretrained checkpoints and then fine-tuned for the seven vending-product classes used in this study: 18 + 0.45 L, Chortoq 0.5 L, Fanta 0.5 L, Moxito 0.5 L, Snickers 80 g, Super Kontik 100 g, and Twix 75 g. The detector-training dataset consisted of annotated vending-cabinet product images collected from the same cabinet environment but separated from the final transaction-level evaluation set. The images were divided into training, validation, and internal test subsets using an 80:10:10 split. All training and inference images were resized to 640 × 640 pixels. The detectors were fine-tuned using supervised object-detection annotations for the seven product classes. Standard data augmentation was applied during training, including image resizing, random horizontal flipping, brightness and contrast adjustment, HSV color jitter, and small geometric perturbations. These augmentations were used to improve robustness against lighting variation, packaging reflections, partial occlusion, and small viewpoint changes inside the cabinet. The validation subset was used to select the detector confidence threshold, NMS threshold, and track-filtering parameters.

During inference, the same confidence threshold of 0.35 was applied to both YOLO11s-seg and RT-DETR-R18 outputs before detector merging. This threshold was shared across all seven product classes; no detector-specific or class-specific confidence thresholds were used. The value of 0.35 was selected because lower thresholds increased false detections caused by reflections, hand occlusion, and shelf re-settling, whereas higher thresholds reduced recall for small confectionery products and partially occluded items. After confidence filtering, detections from the two detectors were merged using class-aware non-maximum suppression with an IoU threshold of 0.45. ByteTrack was applied independently to each camera stream after detector merging. The tracker used the following settings: track_thresh = 0.35, high_thresh = 0.60, match_thresh = 0.80, track_buffer = 30, new_track_thresh = 0.35, fuse_score = true, and mot20 = false. Tracks shorter than 5 selected frames or with mean confidence below 0.35 were treated as unreliable and assigned to the SPURIOUS category before receipt construction. Track-level product class labels were assigned using majority voting over the frame-level detector labels within each track. The exact detector-training configuration was as follows. YOLO11s-seg was fine-tuned for 100 epochs using stochastic gradient descent with an initial learning rate of 0.01, momentum of 0.937, weight decay of 0.0005, and a cosine learning-rate schedule. RT-DETR-R18 was fine-tuned for 100 epochs using AdamW with an initial learning rate of 0.0001, weight decay of 0.0001, and a cosine learning-rate schedule. Detector training was performed on a GPU workstation, whereas the final inference and transaction-recognition runtime evaluation were performed on the Intel N100 CPU-only edge device. This separation reflects the intended deployment scenario: model training is performed offline, while transaction recognition is executed on the edge device.

This configuration makes the detector and tracker stages reproducible and clarifies that the proposed contribution is not a new detector-training method. Instead, the detectors provide frame-level product evidence that is converted into transaction-level decisions by ByteTrack association, zone-aware finite-state event interpretation, and evidence-gated cross-camera fusion.

### 5.2. Ground-Truth Annotation Protocol

Ground-truth labels were created at the transaction-event level rather than only at the frame level. Each transaction was independently reviewed by two human annotators who watched the two camera streams side by side. The annotators recorded the ordered list of customer–product interaction events at the SKU level. Each event was labelled as TAKEN or RETURNED according to whether the product was finally removed from the cabinet or placed back on the shelf. Disagreements between the two annotators were resolved by a third reviewer. The final consolidated annotation contained 227 TAKEN events and 87 RETURNED events, corresponding to 314 transaction-level annotated events. The reported inter-rater agreement was Cohen’s κ = 0.86, indicating a high level of consistency between annotators. This annotation protocol is appropriate for the proposed method because the goal of the system is not simply to detect objects in frames, but to infer product-level transaction events. Therefore, the evaluation compares the final predicted receipt against the event-level ground truth.

### 5.3. Baseline and Compared Configurations

To strengthen the experimental comparison and isolate the contribution of each component, the evaluation includes several detector-level, tracking-level, zone-reasoning-level, and fusion-level baselines (see Table 3). The snapshot-difference baseline is retained because it represents a common endpoint-only recognition strategy used in simple smart vending systems. However, since this baseline does not use temporal information, additional configurations were added to clarify which part of the proposed pipeline is responsible for the observed improvement. The compared configurations are defined as follows.

Snapshot-difference baseline: This method applies product detection to the beginning and ending observation windows of a transaction. The estimated receipt is obtained by comparing the detected product multisets at the start and end of the transaction. This baseline does not use tracking, zone reasoning, or cross-camera event fusion.

YOLO11-seg snapshot only: This configuration uses only YOLO11-seg detections in the snapshot-difference framework. It evaluates whether the segmentation-based detector alone can improve endpoint recognition.

RT-DETR snapshot only: This configuration uses only RT-DETR detections in the snapshot-difference framework. It evaluates whether the transformer-based detector improves endpoint recognition, especially for small or partially occluded products.

Detector ensemble snapshot: This configuration combines YOLO11-seg and RT-DETR outputs using non-maximum suppression but still uses endpoint-only snapshot comparison. It isolates the effect of detector ensembling without temporal tracking or event reasoning.

Camera 0-only tracking: This configuration applies the tracking and event-reasoning pipeline only to Camera 0, which provides a near-perpendicular top-down view. It evaluates the contribution of the perpendicular camera stream alone.

Camera 1-only tracking: This configuration applies the tracking and event-reasoning pipeline only to Camera 1, which observes the cabinet from an approximately 45° convergent view. It evaluates the contribution of the oblique camera stream alone.

ByteTrack with two-zone logic: This configuration introduces continuous temporal association using ByteTrack but uses a simplified two-zone representation. It evaluates the effect of temporal tracking before the full three-zone finite-state model is introduced.

Three-zone FSM without cross-camera fusion: This configuration maps tracks into SHELF, MIDDLE, and EXIT zones and interprets compressed zone sequences using the finite-state event classifier. However, camera outputs are evaluated without the proposed evidence-gated fusion rule. This isolates the contribution of zone-aware finite-state reasoning.

Symmetric union fusion: This configuration accepts any candidate TAKEN or UNKNOWN-supported event from either camera. It represents a permissive fusion strategy and is expected to improve recall but increase false positives.

Intersection-only fusion: This configuration accepts only product-removal events confirmed by both cameras. It represents a conservative fusion strategy and is expected to reduce false positives but miss true pickups when one view is occluded.

Proposed evidence-gated cross-camera fusion: This is the released ZAB-Fusion configuration. It accepts confirmed two-camera TAKEN evidence, admits high-confidence single-camera TAKEN evidence when the opposite camera provides no contradictory event, accepts UNKNOWN evidence only when supported by opposite-camera TAKEN evidence, and rejects isolated weak or ambiguous events.

These configurations allow the evaluation to separate the effects of detector choice, detector ensembling, continuous tracking, zone-aware finite-state reasoning, and cross-camera fusion.

### 5.4. Evaluation Metrics

The system was evaluated at both the event level and the transaction level. At the event level, TAKEN predictions were compared with ground-truth TAKEN events using true positives, false positives, and false negatives. Precision, recall, and F1-score were computed using their standard definitions from TP, FP, and FN. At the transaction level, exact receipt accuracy was computed as the proportion of transactions for which the predicted product classes and quantities exactly matched the ground-truth receipt.

False positives were analyzed separately because they are particularly important in billing systems: a false positive may charge a customer for a product that was not purchased. False negatives were also analyzed because they correspond to missed genuine pickups and operator revenue loss. Therefore, the evaluation reports event-level metrics, false-positive counts, false-negative counts, exact receipt accuracy, and diagnostic error patterns.

Statistical confidence intervals were estimated using transaction-level bootstrap resampling. Specifically, 10,000 bootstrap resamples were generated from the 220 evaluated transactions. The resampling unit was the complete transaction rather than the individual event, so all product events belonging to the same transaction were kept together during resampling. This preserves the dependence structure among multiple TAKEN or RETURNED events within a single customer interaction. For each bootstrap resample, TP, FP, FN, precision, recall, F1-score, and exact receipt accuracy were recomputed. The reported 95% confidence intervals are percentile bootstrap intervals, calculated from the 2.5th and 97.5th percentiles of the bootstrap distribution. No class-based stratification was applied because the evaluation was designed to estimate transaction-level performance under the observed in-service product distribution.

### 5.5. Zone-Boundary Calibration Evaluation

Since the two cameras observe the shelf from different viewpoints, the SHELF–MIDDLE boundary cannot be assumed to be identical for both views. Camera 0 has a near-perpendicular top-down view, while Camera 1 has an approximately 45° convergent view. This causes the same physical shelf region to occupy different vertical image proportions. To evaluate the effect of camera-specific zone calibration, the Camera 1 boundary s_max_ is swept while all other parameters are held fixed. The tested values include the shared boundary and the released asymmetric boundary. The purpose of this ablation is to determine whether asymmetric calibration reduces phantom MIDDLE events without suppressing true pickup events. The cross-camera fusion rule was evaluated using four variants: Camera 0 only, Camera 1 only, symmetric union, and evidence-gated cross-camera fusion. This experiment isolates the effect of decision-level fusion after detection, tracking, and event classification.

1. Camera 0 only: only the perpendicular camera stream is used;
2. Camera 1 only: only the 45° oblique camera stream is used;
3. Symmetric union: any TAKEN or UNKNOWN event from either camera is accepted;
4. Evidence-gated cross-camera fusion: TAKEN events are admitted as strong evidence, while UNKNOWN events require confirmation by a TAKEN event from the opposite camera.

This ablation isolates the contribution of evidence-gated cross-camera fusion. The goal is to test whether the proposed fusion rule can reduce phantom false positives while preserving genuine single-camera evidence. According to the evaluation, evidence-gated cross-camera fusion is the only tested fusion variant that eliminates the phantom-VETO false-positive subset while still admitting genuine single-camera TAKEN evidence.

### 5.6. Runtime Measurement Protocol

To avoid ambiguity between recorded video length and computational latency, the runtime measurement protocol was defined separately from the transaction-video acquisition protocol. Each transaction was recorded using two asynchronous camera streams. The average transaction duration was 27.4 s, corresponding to approximately 800 raw frames per camera. Therefore, a complete transaction contains approximately 1600 raw frames from the two cameras. However, the reported runtime does not correspond to exhaustive detector inference on every raw frame. Instead, the deployed edge-processing mode uses a selected-frame transaction-recognition protocol before detector inference.

The frame-selection protocol was introduced because consecutive video frames in a smart vending transaction are highly redundant. In most parts of the transaction, the shelf state remains unchanged, and product motion occurs only during short intervals when the customer’s hand interacts with the products. Therefore, applying both detectors to all raw frames is computationally inefficient and unnecessary for transaction-level receipt generation. The system first removes lid-opening and lid-closing buffer intervals and then selects motion-relevant frames from the remaining transaction interval. Frame selection is based on temporal spacing and visual-change evidence between neighboring frames. This produces a compact set of representative frames that preserves the product-motion evidence required for tracking and zone-state reasoning.

All selected frames were resized to 640 × 640 pixels before detector inference. The detector ensemble consisted of YOLO11-seg and RT-DETR. Both detectors were executed on an Intel N100 CPU-only edge device. The inference batch size was set to 1, and the same setting was used for both camera streams. CPU execution was performed using four threads. Before recording the runtime values, five warm-up transactions were processed and excluded from the final timing. The reported runtime was then computed as the average over repeated transaction-level measurements on the evaluation set.

The measured runtime includes frame preprocessing, orientation correction, detector inference on selected frames, detector-output merging, non-maximum suppression, ByteTrack association, zone classification, finite-state event interpretation, evidence-gated cross-camera fusion, and receipt construction. Video recording time was not included because it occurs during the customer interaction itself. Raw video file loading and full-frame decoding were also not included in the algorithmic latency because the edge workflow processes the selected transaction frames after the lid-close event. Therefore, the reported runtime represents recognition latency after transaction completion rather than total video acquisition time. Table 4 summarizes the runtime measurement protocol.

Under this protocol, the average processing latency was 562 ms per transaction. This value should not be interpreted as the time required to process all approximately 1600 raw frames with both detectors. Full-frame exhaustive processing would require substantially higher computational cost on CPU-only hardware. Instead, the 562 ms latency corresponds to the practical selected-frame processing mode used for near-real-time post-closure receipt generation.

This clarification is important for interpreting the engineering feasibility of the proposed framework. The system is designed to generate the receipt shortly after the cabinet lid is closed. Since the average transaction lasts 27.4 s and the selected-frame recognition latency is 562 ms, the proposed pipeline can support near-real-time post-closure billing in the evaluated deployment mode. For stricter instant-checkout or exhaustive full-frame video analysis, further acceleration would be required through detector quantization, pruning, adaptive frame selection, single-detector operation, or hardware acceleration using low-power GPU/NPU modules.

### 5.7. Implementation Details and Reproducibility Parameters

To improve reproducibility, this subsection provides the main implementation details and parameter settings used in the evaluation. The proposed framework consists of six modular stages: frame preprocessing, detector inference, detector-output merging, temporal association, zone-aware finite-state event interpretation, and evidence-gated cross-camera fusion. Each camera stream was processed independently until the event-fusion stage.

All video frames were first rotated by 180° to establish a consistent coordinate convention, where the shelf region appears near the upper part of the processed frame and the exit direction appears toward the lower part. The original frame resolution was 1280 × 720 pixels, and both cameras recorded at approximately 29 frames per second. Since Camera 0 and Camera 1 were asynchronous, no frame-level synchronization was assumed. All frames between the lid-opening and lid-closing buffer regions were processed. No sparse frame sampling was used in the reported experiments. The frame-level product detector was implemented as an ensemble of YOLO11-seg and RT-DETR. The two detectors were used because they provide complementary behavior in the cabinet scene: YOLO11-seg provides stable localization for larger bottle-like products, while RT-DETR improves sensitivity to small and partially occluded confectionery products. Both detectors were fine-tuned using the same seven product classes in the vending-cabinet product catalogue: 18 + 0.45 L, Chortoq 0.5 L, Fanta 0.5 L, Moxito 0.5 L, Snickers 80 g, Super Kontik 100 g, and Twix 75 g.

The detector training data were separated from the transaction-level evaluation set. The transaction-level evaluation set contained 220 in-service transactions and was used only for final event-level and receipt-level evaluation. The detector fine-tuning set consisted of annotated cabinet product images extracted from separate training recordings and still images. The training data were divided into training, validation, and internal test subsets using an 80:10:10 split. The same split was used for both YOLO11-seg and RT-DETR fine-tuning to ensure a fair detector-level comparison. For each frame, detections from YOLO11-seg and RT-DETR were first filtered using a confidence threshold of 0.35. The remaining detections were merged using class-aware non-maximum suppression with an IoU threshold of 0.45. The resulting detection set was then passed to ByteTrack for temporal association. ByteTrack was applied independently to the two camera streams. Tracks shorter than five frames were removed from the billing decision stage and classified as SPURIOUS. In addition, tracks with a mean confidence lower than 0.35 were also classified as SPURIOUS. The product class of each track was assigned using majority voting over the frame-level class labels in the track.

For zone-aware event interpretation, each bounding box observation was mapped to one of three vertical cabinet zones: SHELF, MIDDLE, or EXIT. The normalized bottom-edge coordinate was used:

$$y_{norm}=\frac{y_{2}}{H},$$

where y_2 is the bottom coordinate of the bounding box and H is the image height. The zone assignment was defined as follows:

*SHELF*, *if y_norm_* < *s_max_*

*MIDDLE, if s_max_* ≤ *y_norm_* < *m_max_*

*EXIT*, *if y_norm_* ≥ *m_max_*

Because the two cameras observe the cabinet from different viewpoints, camera-specific zone boundaries were used. For Camera 0, which provides a near-perpendicular top-down view, the values were *s_max_* = 0.62 and *m_max_* = 0.82. For Camera 1, which observes the shelf from an approximately 45° convergent angle, the values were *s_max_* = 0.65 and *m_max_* = 0.82. These asymmetric thresholds reduce false MIDDLE-zone assignments in the oblique view.

Each raw zone sequence was compressed by removing consecutive repeated labels. The compressed sequence was then interpreted using a finite-state event classifier. The following rules were used:

[*SHELF*, *MIDDLE*, *EXIT*] → *TAKEN* [*SHELF*, *EXIT*] → *TAKEN* [*SHELF*, *MIDDLE*, *SHELF*] → *RETURNED* [*SHELF*, *EXIT*, *SHELF*] → *INSPECTED* [*MIDDLE*] → *UNKNOWN* [*EXIT*] → *SPURIOUS* [*SHELF*] → *SPURIOUS* *Track length* < 5 *frames* → *SPURIOUS* *Mean track confidence* < 0.35 → *SPURIOUS*

The camera-specific event streams were then integrated using evidence-gated cross-camera fusion. We use the term evidence-gated cross-camera fusion because the rule is conservative but not a pure intersection rule. The fusion rule distinguishes four cases. First, if both cameras produce a TAKEN event for the same product class, the event is accepted as confirmed two-camera evidence. Second, if one camera produces a high-confidence TAKEN event and the other camera provides no contradictory event for the same product class, the event is accepted as admitted single-camera evidence. Third, an UNKNOWN event is accepted only if the opposite camera produces a TAKEN event for the same product class in the same transaction. Fourth, isolated UNKNOWN events, isolated SPURIOUS events, and contradictory ambiguous events are rejected.

The final receipt quantity for each class c is computed from the admitted TAKEN events:

$$q_{c}=\;N_{admitted\left(TAKEN,\;c\right)},$$

where q_c_ denotes the estimated number of products of class c removed from the cabinet. This rule ensures that each billed item can be traced back to its camera source, track identifier, product class, compressed zone sequence, event label, and fusion decision. Table 5 summarizes the main implementation parameters used in the evaluation.

These details make the proposed pipeline reproducible at the level of detector inference, tracking, zone assignment, event interpretation, and cross-camera fusion. They also clarify that the main contribution of ZAB-Fusion is not a new detector architecture, but the transformation of frame-level product detections into auditable transaction-level events under sensorless dual-camera cabinet constraints.

## 6. Results and Discussion

This section presents and discusses the experimental results of the proposed ZAB-Fusion framework. The analysis includes overall recognition performance, the calibration trajectory from the snapshot-difference baseline to the released configuration, the effects of asymmetric zone calibration and evidence-gated cross-camera fusion, runtime behavior on CPU-only edge hardware, residual failure cases, and the practical implications of the proposed framework. The first experiment compares the released ZAB-Fusion configuration with the snapshot-difference baseline. The baseline uses only the beginning and ending observation windows of each transaction, whereas ZAB-Fusion uses continuous product tracking, zone-aware event reasoning, and evidence-gated cross-camera fusion. The evaluation was performed on 220 in-service transactions containing 227 ground-truth TAKEN events and 87 RETURNED events. Table 6 compares the snapshot-difference baseline with the released ZAB-Fusion configuration. The baseline uses only the beginning and ending observation windows of the transaction, while ZAB-Fusion uses continuous product tracking, zone-aware finite-state interpretation, and evidence-gated cross-camera fusion.

The confidence intervals in Table 6 were computed using the transaction-level bootstrap procedure described in Section 5.4. Because the bootstrap unit was the complete transaction, multi-event transactions were resampled as intact units rather than as independent product events. This avoids treating product events from the same customer interaction as statistically independent observations.

The results show that ZAB-Fusion substantially improves transaction-level product recognition compared with endpoint-only snapshot reasoning. Recall increased from 0.665 to 0.925, and F1-score increased from 0.780 to 0.944. Importantly, this improvement was achieved without increasing the billing-oriented false-positive risk. Precision increased slightly from 0.944 to 0.963, and the number of false-positive TAKEN events decreased from 9 to 8. In a customer-facing billing system, false positives are particularly important because they may result in an incorrect charge. Therefore, the proposed method is not evaluated only by F1-score. We also analyze false-positive frequency and exact receipt accuracy. ZAB-Fusion produced 8 false-positive TAKEN events over 220 transactions, corresponding to 0.036 false-positive events per transaction. In other words, fewer than four false billing candidates occurred per 100 transactions in the evaluation. At the same time, missed genuine pickups decreased from 76 with the snapshot-difference baseline to 17 with ZAB-Fusion. This confirms that the proposed motion-aware reasoning improves pickup recovery while maintaining a low false-positive rate.

To assess metric uncertainty, 95% confidence intervals were computed using transaction-level resampling. For ZAB-Fusion, precision was 0.963 with an approximate 95% interval of 0.929–0.981, recall was 0.925 with an approximate 95% interval of 0.883–0.953, and exact receipt accuracy was 0.900 with an approximate 95% interval of 0.853–0.933.

### 6.1. Calibration Trajectory from Snapshot-Difference to ZAB-Fusion

To understand how each design component contributes to the result, the system was evaluated through a four-step calibration trajectory. Each version changes one main design element relative to the previous version (Table 7). The evaluated configurations are:

v1—snapshot-difference baseline;
v2—ByteTrack continuous tracking with simplified two-zone logic;
v3—three-zone finite-state machine with evidence-gated cross-camera fusion;
v4—released ZAB-Fusion with asymmetric per-camera zone calibration.

**Table 7:** Calibration trajectory from snapshot-difference baseline to released ZAB-Fusion configuration.

Version	Main Configuration	TP	FP	FN	Precision	Recall	F1-Score
v1	Snapshot-difference baseline	151	9	76	0.944	0.665	0.780
v2	ByteTrack continuous tracking, two-zone logic	209	25	18	0.893	0.921	0.907
v3	Three-zone FSM + evidence-gated fusion	203	12	24	0.944	0.894	0.918
v4	Full ZAB-Fusion with asymmetric zone calibration	210	8	17	0.963	0.925	0.944

The transition from v1 to v2 shows the benefit of continuous tracking. Recall increased from 0.665 to 0.921 because the system was no longer limited to endpoint snapshots. However, this improvement came with an increase in false positives, from 9 to 25, because tracking also preserved hand-induced movements, shelf re-settling artifacts, and ambiguous short trajectories. The transition from v2 to v3 shows the effect of three-zone finite-state reasoning and evidence-gated cross-camera fusion. Precision increased from 0.893 to 0.944, and false positives decreased from 25 to 12. This confirms that symbolic event reasoning suppresses many ambiguous trajectories that are produced by tracking alone. The final v4 configuration adds asymmetric camera-specific zone calibration. This configuration achieves the best overall performance, with precision of 0.963, recall of 0.925, and F1-score of 0.944. It also produces the lowest number of false positives among the tracking-based configurations. Therefore, v4 is preferred not only because it achieves the highest F1-score, but also because it provides the most appropriate billing-oriented operating point.

The evidence-gated cross-camera fusion rule was evaluated using four variants: Camera 0 only, Camera 1 only, symmetric union, and evidence-gated cross-camera fusion. This experiment isolates the effect of decision-level fusion after detection, tracking, and event classification (Table 8).

The single-camera variants show that each view provides useful but incomplete evidence. Camera 0 achieved precision of 0.901 and recall of 0.841, while Camera 1 achieved precision of 0.885 and recall of 0.815. Camera 0 performed slightly better because its near-perpendicular view is less affected by perspective distortion, whereas Camera 1 provides complementary oblique evidence but is more sensitive to shelf-depth ambiguity and occlusion. Symmetric union achieved the highest recall of 0.943 because it accepts events from either camera, but it also produced 29 false positives, including 18 phantom false positives. This behavior is undesirable for billing because false positives may lead to incorrect customer charges. Intersection-only fusion achieved the highest precision of 0.972 but reduced recall to 0.767 because it rejected genuine pickups that were visible in only one camera. The proposed evidence-gated cross-camera fusion achieved precision of 0.963, recall of 0.925, and F1-score of 0.944. It admitted 36 genuine single-camera true-positive events while reducing false positives to 8 and eliminating phantom false positives in the evaluated dataset. This confirms that evidence-gated fusion provides a more suitable billing-oriented operating point than either symmetric union or strict intersection.

To determine which part of the proposed pipeline contributes to the final improvement, we evaluated detector-level, tracking-level, zone-reasoning-level, and fusion-level baselines. The comparison is shown in Table 9.

The expanded comparison shows that the final improvement is not caused by detector ensembling alone. YOLO11-seg-only and RT-DETR-only snapshot variants improve the endpoint baseline only modestly, and the detector ensemble snapshot still remains limited because it cannot observe intermediate product motion. This confirms that the main limitation of snapshot-based recognition is not only detector accuracy, but also the absence of temporal event semantics. Single-camera tracking improves recall compared with snapshot recognition, but each camera has different weaknesses. Camera 0 provides a more stable top-down view, while Camera 1 provides complementary oblique evidence but is more affected by perspective distortion and shelf-depth ambiguity. This supports the use of two cameras as independent visual witnesses rather than relying on a single view. The comparison between ByteTrack with two-zone logic and three-zone FSM reasoning shows the value of explicit zone-aware event interpretation. Tracking alone preserves more candidate movements, but it also increases false positives caused by hand-induced motion, shelf re-settling, and short ambiguous tracks. The three-zone finite-state model suppresses many of these noisy trajectories by distinguishing TAKEN, RETURNED, INSPECTED, UNKNOWN, and SPURIOUS events.

The fusion comparison further clarifies the role of the proposed evidence-gated cross-camera fusion rule. Symmetric union achieves high recall but increases the risk of false positives, which is undesirable in a billing application. Intersection-only fusion achieves high precision but suppresses genuine pickups when one camera is occluded or fails to generate a complete track. The proposed evidence-gated fusion achieves the best overall balance, with precision of 0.963, recall of 0.925, and F1-score of 0.944. This confirms that the proposed framework improves transaction recognition through the combined effect of temporal tracking, zone-aware finite-state reasoning, and auditable cross-camera evidence gating.

### 6.2. Diagnostic Analysis: Transaction-Level, Per-Class, and Error-Case Evaluation

Aggregate metrics such as precision, recall, and F1-score provide an overall view of system performance, but they do not fully explain how a smart vending recognition system behaves at the receipt level. Since the final output of the proposed system is a transaction receipt rather than an isolated object detection result, we provide additional diagnostic analysis at the transaction, product-class, and error-case levels (see Table 10). A transaction was considered exactly correct when the predicted product classes and quantities matched the ground-truth receipt for the entire transaction. A partially correct transaction indicates that at least one product was correctly identified, but the receipt contained either a missed item, an extra item, or a wrong product class. An incorrect transaction indicates that the predicted receipt did not match the ground truth in a practically useful way.

The snapshot-difference baseline generated completely correct receipts for 142 of 220 transactions, corresponding to an exact receipt accuracy of 0.645. In contrast, the proposed ZAB-Fusion configuration generated completely correct receipts for 198 of 220 transactions, increasing exact receipt accuracy to 0.900. This result is important because the final commercial output of a smart vending system is the transaction receipt. Therefore, receipt-level accuracy provides a more application-oriented evaluation than event-level F1-score alone. The improvement in receipt accuracy shows that explicit motion semantics reduce the number of transactions affected by missed pickups or ambiguous product movements. The baseline often failed when a product was removed during the middle of the transaction but was not clearly represented in the endpoint frames. ZAB-Fusion reduced this failure mode by using continuous tracking, zone-aware finite-state interpretation, and evidence-gated cross-camera fusion.

To make the evaluation more transparent, Table 11 presents representative transaction-level examples. Each row shows the ground-truth receipt, the output of the snapshot-difference baseline, the output of ZAB-Fusion, and the corresponding diagnostic interpretation.

These representative cases show that ZAB-Fusion improves recognition mainly in transactions involving small products, temporary occlusions, pickup–return actions, and intermediate product motion. The baseline performs well when the initial and final product states are clearly visible, but it fails when the decisive transaction evidence occurs between the endpoint frames.

To avoid overstating the available per-class evidence, Table 12 reports class-specific diagnostic observations rather than quantitative per-class performance metrics. The quantitative event-level performance of the complete system is reported at the overall level using TP, FP, FN, precision, recall, and F1-score. The per-class table is used only to summarize the dominant qualitative error patterns observed for each product class, such as small-object miss detection, occlusion sensitivity, visual similarity, and shelf re-settling effects. The seven product classes differ in size, shape, packaging appearance, and visibility in the two camera views. Large bottle-like products are generally easier to localize and track, whereas small confectionery products are more sensitive to occlusion, blur, and class confusion. Table 12 summarizes the per-class diagnostic patterns observed in the evaluation.

Table 12 should be interpreted as a qualitative diagnostic summary. It identifies which product classes were more affected by small object size, partial hand occlusion, packaging similarity, or shelf re-settling. The table is not intended to replace quantitative per-class evaluation. The overall quantitative results are reported in above sections, where event-level TP, FP, FN, precision, recall, F1-score, and transaction-level exact receipt accuracy are provided. The per-class analysis confirms that most remaining errors occur for small confectionery products. These products occupy a smaller region in the image than bottles and are more likely to be occluded by the customer’s hand during pickup. In addition, Twix 75 g and Super Kontik 100 g have similar size and shelf placement, which can lead to class confusion when the product is visible for only a few frames. The most frequent class-confusion pattern occurred between Twix 75 g and Super Kontik 100 g. This confusion is caused by three factors. First, both products are small confectionery items and occupy fewer pixels than beverage bottles. Second, they are often placed in nearby shelf positions. Third, during customer interaction, they may be partially covered by the hand or by neighboring products. Under these conditions, frame-level detectors sometimes produce inconsistent class labels along the same track. ZAB-Fusion reduces this problem in two ways. First, track-level class assignment is based on majority voting over all frame-level detections in the track rather than on a single frame. Second, event-level fusion can use complementary evidence from the second camera when one camera produces an unstable class prediction. However, confusion between visually similar small products remains one of the main residual error modes. Future work will address this issue using stronger fine-grained product embeddings, hand–object interaction constraints, and trajectory-level instance separation. The remaining errors were grouped into three diagnostic categories: true positives, false positives, and false negatives.

True-positive cases: True positives usually occurred when a product trajectory showed a clear movement from the SHELF zone through the MIDDLE zone toward the EXIT zone. In these cases, the compressed zone sequence was typically [SHELF, MIDDLE, EXIT] or [SHELF, EXIT]. These trajectories were reliably classified as TAKEN by the finite-state event classifier. The evidence was strongest when both cameras observed the product motion, but ZAB-Fusion could also admit high-confidence single-camera TAKEN evidence when the opposite view was temporarily occluded.

False-positive cases: False positives mainly occurred when a non-purchased product moved because of shelf re-settling or hand-induced contact. For example, when a customer removed a neighboring product, another product could shift from the SHELF zone toward the MIDDLE zone and then return to the shelf. If this movement was only partially observed, the resulting track could resemble a TAKEN event. Evidence-gated fusion reduced this problem by rejecting isolated UNKNOWN events and ambiguous tracks, but a small number of false positives remained.

False-negative cases: False negatives mainly occurred for small products that were visible for only a short time during removal. Snickers 80 g, Twix 75 g, and Super Kontik 100 g were more likely to be missed than larger, bottle-like products. These errors were often associated with hand occlusion, motion blur, short track duration, or low detector confidence. Some false negatives were caused by tracks being filtered as SPURIOUS because they were shorter than five frames or had mean confidence below 0.35.

Overall, the diagnostic analysis shows that ZAB-Fusion improves transaction recognition by converting intermediate product motion into auditable event evidence. The remaining errors are mainly associated with small-product visibility, visually similar packaging, shelf re-settling, and hand occlusion. These findings motivate future extensions involving hand–object interaction modeling, fine-grained product re-identification, and improved tracking of multiple identical items.

### 6.3. Runtime Performance and Real-Time Feasibility

The runtime performance of the proposed ZAB-Fusion framework was evaluated on an Intel N100 CPU-only edge device. Since the target application is a smart vending cabinet, it is important to distinguish between three different time-related quantities: the recorded transaction duration, the number of raw frames generated by the cameras, and the computational latency required to generate the receipt after the transaction is completed.

In the evaluated dataset, the average transaction duration was 27.4 s. At approximately 29 frames per second, this corresponds to about 800 raw frames per camera and approximately 1600 raw frames for the two-camera transaction. However, the proposed deployment mode does not apply YOLO11-seg and RT-DETR exhaustively to every raw frame. Instead, ZAB-Fusion uses a selected-frame transaction-processing protocol. After removing lid-opening and lid-closing buffer intervals, the system selects motion-relevant frames using temporal spacing and visual-change evidence. Detector inference is then applied only to the selected frames, while redundant neighboring frames are not processed by the detector ensemble.

Under this selected-frame protocol, the average number of frames used for detector inference was 8 frames per camera, corresponding to 16 selected frames per complete two-camera transaction. All selected frames were resized to 640 × 640 pixels before detector inference. The runtime reported in this study therefore represents the wall-clock latency of the selected-frame transaction-recognition configuration, not exhaustive full-frame processing of all approximately 1600 raw frames.

Table 13 shows the runtime breakdown. The total average processing latency was 562 ms per transaction. This includes frame preprocessing, orientation correction, detector inference on selected frames, detector-output merging, non-maximum suppression, ByteTrack association, zone classification, finite-state event interpretation, evidence-gated cross-camera fusion, and receipt construction. Video acquisition time was not included because it occurs during the customer interaction itself. Exhaustive raw-frame decoding was also not included because the deployed workflow operates on selected transaction frames after the lid-close event.

The runtime results show that detector inference is the dominant computational component. YOLO11-seg and RT-DETR together required 462 ms per transaction, corresponding to 82.2% of the total runtime. In contrast, the proposed symbolic reasoning stages, including zone classification, finite-state event interpretation, and evidence-gated cross-camera fusion, required only 3 ms in total. ByteTrack association required 12 ms. These results indicate that the proposed motion-semantics layer adds negligible computational overhead compared with detector inference. The reported 562 ms latency should therefore be interpreted carefully. It does not mean that the Intel N100 CPU-only device processed all approximately 1600 raw frames with two deep detectors in less than one second. Full-frame exhaustive inference over every frame from both cameras would require substantially higher computational cost and is not the deployment mode evaluated in this study. The selected-frame protocol is an essential part of the edge-processing design because transaction-level receipt generation depends on representative product-motion evidence rather than on redundant frame-by-frame analysis. From a practical deployment perspective, the proposed framework supports near-real-time post-closure receipt generation. In the evaluated workflow, the customer opens the cabinet, interacts with the products, closes the lid, and then the selected-frame recognition pipeline generates the receipt. Since the average recorded transaction duration is 27.4 s and the average post-closure processing latency is 562 ms, the receipt can be generated shortly after the transaction ends. This latency is suitable for asynchronous post-transaction billing and near-real-time customer receipt generation in the evaluated cabinet setting.

However, the current implementation should not be interpreted as a full real-time video analytics system that processes every incoming frame with both detectors during the transaction. Stricter instant-checkout scenarios, continuous frame-level monitoring, or larger product catalogues may require additional acceleration. Future optimization will therefore focus on detector quantization, pruning, adaptive frame selection, motion-triggered inference, single-detector operation under stable conditions, and hardware acceleration using low-power GPU or NPU modules. These improvements are expected to reduce latency further while preserving the interpretability and auditability of the proposed zone-aware event-reasoning framework.

### 6.4. Failure Case Diagnostics

To understand the remaining errors in the released configuration, all false positives were manually inspected (see Table 14). ZAB-Fusion v4 produced 8 false-positive TAKEN events over 220 transactions. These errors were mainly associated with passive shelf re-settling, hand-induced product motion, short unstable tracks, and class confusion between visually similar small confectionery products. These false positives belong to three different categories. The first false positive was a phantom MIDDLE track. Its compressed zone sequence was [MIDDLE], so the finite-state classifier labelled it as UNKNOWN. The event was later admitted because the opposite camera independently produced a TAKEN event of the same class. The root cause was partial occlusion and detector instability around a product hidden behind another item. The second false positive was a class-flip between visually similar confectionery products, especially Twix and Super Kontik. The trajectory itself was correctly classified as TAKEN, but the track-level majority class label was wrong because the detector confidence margin between the two classes was small. The third false positive was a short ghost track near the exit region. Although the track barely passed the length and confidence thresholds, it was paired with an incorrect event from the opposite camera and therefore survived the fusion process. These errors indicate that the remaining false positives are not caused by one systematic weakness of the FSM. Instead, they arise from different sources: detector misclassification, partial occlusion, unstable track generation, and incorrect cross-camera confirmation. Therefore, future improvements should target detector retraining, hard-negative mining, hand–product interaction gating, and track-level fusion. The false negatives are mostly detection-level failures. If the detector fails to produce enough valid observations for a small product, ByteTrack cannot generate a stable track, and the downstream zone classifier has no trajectory to interpret. This is especially problematic for small confectionery items such as Snickers, Twix, and Super Kontik, which may occupy only approximately 25–40 pixels in height in some views. Thus, ZAB-Fusion improves decision-level reasoning, but it cannot fully compensate for missing or consistently incorrect detector outputs. The framework should therefore be considered complementary to detector retraining rather than a replacement for it.

Direct numerical comparison with previously published retail vision systems is difficult because datasets, cabinet geometry, product catalogues, sensing hardware, annotation protocols, and billing definitions differ substantially across studies. Therefore, instead of reporting non-comparable performance values, this section provides a contextual comparison of system characteristics.

This table is intended as a contextual comparison rather than a direct benchmark ranking. The proposed method differs from static recognition and snapshot-difference approaches because it explicitly models product motion. It also differs from generic multi-object tracking because it converts trajectories into auditable transaction-level events. Since published systems are evaluated under different datasets and operating assumptions, direct numerical comparison is not reported here.

### 6.5. Limitations and Threats to Validity

Several limitations should be acknowledged. First, although the evaluation dataset contains 220 in-service transactions, 227 ground-truth TAKEN events, and 87 RETURNED events, it was collected from one cabinet and one deployment site. Therefore, the results should be interpreted as single-site in-service validation rather than definitive evidence of general performance across all smart vending environments. Lighting conditions, camera placement, cabinet geometry, product arrangement, customer behavior, condensation patterns, and packaging appearance may vary across deployments. Broader validation using multiple cabinet models, different camera placements, more diverse product layouts, and multiple store environments is required before making stronger claims about general commercial robustness. Second, the current evaluation does not systematically cover all challenging operating conditions that may occur in practical vending deployments. These include dim lighting, severe internal condensation, strong reflections, extreme hand occlusion, highly cluttered shelf layouts, and rapid consecutive interactions. Although the collected transactions include real customer interactions, pickup–return cases, inspection actions, occlusion, and shelf re-settling events, the coverage of such difficult conditions remains limited. Future experiments should therefore include controlled stress tests under challenging illumination, reflection, condensation, occlusion, and dense-product-placement conditions. Third, the current framework does not explicitly model the customer’s hand or hand–object contact. ZAB-Fusion infers product-removal events from product tracks and zone transitions. The finite-state event classifier suppresses many non-purchase trajectories by assigning static shelf tracks, short tracks, low-confidence tracks, returned products, inspected products, and isolated ambiguous tracks to non-billing categories. However, false positives may still occur when an untouched product moves passively because of shelf re-settling, contact with a neighboring product, or indirect hand-induced motion. Since not every product movement corresponds to a customer purchase, future versions should incorporate hand detection, hand–product proximity constraints, contact-state estimation, and temporal causality analysis between hand motion and product motion. Such extensions could help distinguish active product removal from passive product displacement. Fourth, the current evidence-gated fusion rule operates primarily at the accepted event and product-class quantity level. Each accepted event retains its camera source, track identifier, product class, compressed zone sequence, event label, confidence score, and fusion decision for auditability. However, the present implementation does not fully resolve instance identity when multiple identical products are removed in close temporal proximity. If two visually identical products are taken consecutively or simultaneously under severe occlusion, fragmented or overlapping tracks may lead to undercounting or overcounting. Future work should therefore investigate trajectory-level instance separation, cross-view track matching, temporal spacing constraints, and product-instance re-identification. Fifth, the framework depends on the quality of detector and tracker outputs. Although ZAB-Fusion improves transaction recognition by converting product tracks into auditable motion events, missed detections, fragmented tracks, identity switches, and class confusion can still affect the final receipt. These issues are especially relevant for small confectionery products and visually similar packaging. Future work will optimize the detection and tracking modules through small-product detector fine-tuning, fine-grained SKU representation learning, stronger temporal association, and improved cross-camera track consistency.

Finally, lightweight optimization remains important for broader deployment. Runtime analysis shows that the average processing latency is 562 ms per transaction on the Intel N100 CPU-only edge device, and the symbolic reasoning stages require only 3 ms. Therefore, the current implementation is compatible with near-real-time post-closure receipt generation in the evaluated workflow. However, stricter instant-checkout scenarios may require lower latency. Future optimization will focus on detector quantization, pruning, motion-triggered frame selection, adaptive frame skipping, single-detector operation under stable conditions, and hardware acceleration using low-power GPU or NPU modules. These improvements are expected to further reduce latency while preserving the interpretability and auditability of the proposed zone-aware event-reasoning framework.

## 7. Conclusions

This paper presented ZAB-Fusion, a dual-camera edge sensing framework with zone-aware multi-object tracking for sensorless smart vending cabinets. The proposed framework addresses the limitations of endpoint-based snapshot-difference recognition, which compares only the initial and final transaction frames and therefore cannot explicitly represent intermediate product motion, such as pickup, return, inspection, temporary occlusion, and shelf re-settling. ZAB-Fusion transforms frame-level product detections into auditable transaction-level events. The framework combines YOLO11-seg and RT-DETR detection, ByteTrack temporal association, a three-zone vertical cabinet model, finite-state event interpretation, and evidence-gated cross-camera fusion. Each product trajectory is converted into a symbolic zone sequence and classified as TAKEN, RETURNED, INSPECTED, UNKNOWN, or SPURIOUS. The camera-specific event streams are then fused to generate the final product-level receipt.

The framework was evaluated on 220 in-service transactions containing 227 ground-truth TAKEN events and 87 RETURNED events across seven product classes. Compared with the snapshot-difference baseline, ZAB-Fusion improved recall from 0.665 to 0.925 and F1-score from 0.780 to 0.944, while maintaining high precision of 0.963. At the transaction level, exact receipt accuracy increased from 0.645 to 0.900. Runtime analysis on an Intel N100 CPU-only edge device showed an average processing latency of 562 ms per transaction under the selected-frame inference protocol. This result supports near-real-time post-closure receipt generation in the evaluated deployment mode, but it should not be interpreted as exhaustive processing of all raw frames in both camera streams. Full-frame processing or stricter instant-checkout scenarios would require further detector optimization, adaptive frame selection, or hardware acceleration. These results show that explicit motion semantics and auditable cross-camera fusion can improve transaction-level recognition in sensorless smart vending cabinets without adding RFID tags, weight sensors, shelf load cells, or other product-level sensing hardware. The main contribution of this work is not a new detector architecture, but an interpretable framework that converts visual product trajectories into receipt-level decisions under realistic dual-camera cabinet constraints.

The current framework also has two important limitations related to industrial deployment. First, the fusion rule counts accepted product-removal events primarily at the product-class level and does not yet provide complete instance-level separation for multiple identical products removed consecutively under severe occlusion. Second, the framework does not explicitly model hand–object interaction, which limits its ability to distinguish genuine product removal from passive shelf movement or indirect product displacement. Future work will address these issues by integrating trajectory-level instance separation, cross-view track matching, product-instance re-identification, hand detection, hand–product contact modeling, and temporal causality constraints. These extensions are necessary for improving robustness in dense product layouts and for moving closer to a complete industrial billing solution.

## Figures and Tables

**Table 1:** Finite-state event classification rules.

Compressed Zone Sequence	Event Label	Interpretation
[SHELF, MIDDLE, EXIT]	TAKEN	Product is picked up and removed from the cabinet.
[SHELF, EXIT]	TAKEN	Fast pickup; the MIDDLE zone is skipped due to motion blur or sparse detections.
[SHELF, MIDDLE, SHELF]	RETURNED	Product is lifted and placed back on the shelf.
[SHELF, EXIT, SHELF]	INSPECTED	Product is moved toward the exit region but returned.
[MIDDLE]	UNKNOWN	Track starts in the middle of the trajectory; evidence is incomplete.
[EXIT]	SPURIOUS	Ghost track or detection near the exit region.
[SHELF]	SPURIOUS	Static product that did not move.
Track length < 5	SPURIOUS	Short unreliable track.
Mean confidence < 0.35	SPURIOUS	Low-confidence unreliable track.

**Table 2:** Summary of the in-service validation dataset.

Property	Value	Description
Number of transactions	220	Real in-service customer transactions
Cameras per transaction	2	Camera 0 and Camera 1
Camera synchronization	Asynchronous	No frame-level synchronization assumed
Resolution	1280 × 720	Native sensor output
Frame rate	Approximately 29 fps	For both camera streams
Average transaction duration	27.4 s	After removing lid-open/close buffers
Frames per camera per transaction	Approximately 800	Depends on transaction duration
Product classes	7	Beverage bottles and confectionery items
Ground-truth TAKEN events	227	Product pickups
Ground-truth RETURNED events	87	Pick-and-place-back interactions
Total annotated events	314	TAKEN + RETURNED events

**Table 3:** Compared configurations used in the experimental evaluation.

Version	Configuration	Main Purpose
v1	Snapshot-difference baseline	Endpoint-only recognition
v2	ByteTrack continuous tracking with two-zone logic	Effect of temporal tracking
v3	Three-zone FSM + evidence-gated fusion	Effect of event reasoning and conservative fusion
v4	Full ZAB-Fusion with asymmetric zone calibration	Released proposed system

**Table 4:** Runtime measurement protocol.

Item	Setting Used in This Study
Edge device	Intel N100 CPU-only device
Camera streams	Two asynchronous streams
Average transaction duration	27.4 s
Raw frames per camera	Approximately 800
Raw frames per transaction	Approximately 1600
Exhaustive full-frame inference	Not used
Frame-selection strategy	Motion-relevant selected-frame processing
Average selected frames per camera	8
Average selected frames per transaction	16
Detector input size	640 × 640 pixels
Detector ensemble	YOLO11-seg + RT-DETR
Batch size	1
CPU threads	4
Warm-up runs	5 transactions
Runtime unit	Wall-clock latency per transaction
Included in timing	Preprocessing, detector inference on selected frames, NMS, tracking, zone/FSM/fusion, receipt construction
Excluded from timing	Video acquisition time and exhaustive raw-frame decoding

**Table 5:** Implementation details and reproducibility parameters.

Component	Parameter	Value Used in This Study
Input video	Resolution	1280 × 720 pixels
Input video	Frame rate	Approximately 29 fps
Input video	Camera streams	Two asynchronous streams
Preprocessing	Orientation correction	180° rotation before processing
Frame selection	Sampling strategy	All frames after lid-open/lid-close buffer removal
Product classes	Number of classes	7
Detector 1	Model	YOLO11-seg
Detector 2	Model	RT-DETR
Detector training	Fine-tuning	Fine-tuned on cabinet product images
Data split	Train/validation/test	80:10:10
Detection filtering	Confidence threshold	0.35
Detection merging	NMS type	Class-aware NMS
Detection merging	NMS IoU threshold	0.45
Temporal association	Tracker	ByteTrack
Track class assignment	Rule	Majority vote over frame-level labels
Zone coordinate	Coordinate used	Normalized bounding-box bottom edge, y_norm = y_2/H
Camera0 zone boundary	s_max_/m_max_	0.62/0.82
Camera1 zone boundary	s_max_/m_max_	0.65/0.82
FSM output labels	Event classes	TAKEN, RETURNED, INSPECTED, UNKNOWN, SPURIOUS
Fusion module	EGCCF name	Evidence-gated cross-camera fusion
Final output	Receipt rule	Count admitted TAKEN events per class
Hardware	Edge device	Intel N100 CPU-only device, 8 GB RAM

**Table 6:** Overall comparison between snapshot-difference baseline and released ZAB-Fusion.

Method	TP	FP	FN	Precision	Recall	F1-Score
Snapshot-difference baseline	151	9	76	0.944	0.665	0.780
ZAB-Fusion v4	210	8	17	0.963	0.925	0.944

**Table 8:** Ablation of evidence-gated cross-camera fusion rule.

Fusion Variant	TP	TP_single_	FP	FP_phantom_	FN	Precision	Recall	F1-Score
Camera 0 only	191	N/A	21	9	36	0.901	0.841	0.870
Camera 1 only	185	N/A	24	12	42	0.885	0.815	0.849
Symmetric union	214	48	29	18	13	0.881	0.943	0.911
Intersection-only fusion	174	0	5	1	53	0.972	0.767	0.857
Evidence-gated cross-camera fusion	210	36	8	0	17	0.963	0.925	0.944

**Table 9:** Expanded baseline and component ablation comparison.

Method	Precision	Recall	F1-Score	Main Observation
Snapshot-difference baseline	0.944	0.665	0.780	Conservative endpoint-only recognition; many true pickups are missed
YOLO11-seg snapshot only	0.931	0.696	0.796	Stable for larger bottle-like products but still endpoint-limited
RT-DETR snapshot only	0.918	0.714	0.803	More sensitive to small products but still lacks motion evidence
Detector ensemble snapshot	0.927	0.744	0.825	Detector ensembling improves endpoint recognition but cannot model pickup/return motion
Camera 0 only + tracking	0.902	0.842	0.871	Perpendicular view is stable but sensitive to hand occlusion
Camera 1 only + tracking	0.884	0.815	0.848	Oblique view provides complementary evidence but introduces more geometric ambiguity
ByteTrack + two-zone logic	0.893	0.921	0.907	Tracking improves recall but introduces more false positives
Three-zone FSM without fusion	0.922	0.890	0.906	Zone-aware event reasoning suppresses some noisy trajectories
Symmetric union fusion	0.881	0.943	0.911	High recall but less suitable for billing because false positives increase
Intersection-only fusion	0.972	0.767	0.857	Very conservative; misses true pickups when one camera is occluded
Proposed evidence-gated fusion	0.963	0.925	0.944	Best balance between recall and billing-oriented precision

**Table 10:** Transaction-level receipt accuracy.

Method	Correct Receipts	Partially Correct Receipts	Incorrect Receipts	Exact Receipt Accuracy
Snapshot-difference baseline	142/220	51/220	27/220	0.645
ZAB-Fusion v4	198/220	17/220	5/220	0.900

**Table 11:** Representative transaction-level diagnostic examples.

Transaction Type	Ground Truth	Snapshot-Difference Output	ZAB-Fusion Output	Diagnostic Interpretation
Clean single-item pickup	Fanta 0.5 L × 1	Fanta 0.5 L × 1	Fanta 0.5 L × 1	Both methods correct
Small-item pickup	Snickers 80 g × 1	Empty receipt	Snickers 80 g × 1	ZAB-Fusion recovers small-item motion missed by endpoint comparison
Multiple-item pickup	Moxito 0.5 L × 1; Twix 75 g × 1	Moxito 0.5 L × 1	Moxito 0.5 L × 1; Twix 75 g × 1	Baseline misses the small confectionery item
Pickup and return	Chortoq 0.5 L returned	Chortoq 0.5 L × 1	Empty receipt	ZAB-Fusion correctly suppresses returned item
Inspection without purchase	Super Kontik 100 g inspected	Super Kontik 100 g × 1	Empty receipt	Zone-aware FSM prevents inspection from being billed
Shelf re-settling after pickup	Fanta 0.5 L × 1	Fanta 0.5 L × 1; Moxito 0.5 L × 1	Fanta 0.5 L × 1	Evidence-gated fusion suppresses false positive caused by shelf movement
Visually similar products	Twix 75 g × 1	Super Kontik 100 g × 1	Twix 75 g × 1	Tracking and multi-view evidence reduce class confusion
Strong occlusion	Moxito 0.5 L × 1	Empty receipt	Moxito 0.5 L × 1	Single-view TAKEN evidence admitted when opposite view is occluded

**Table 12:** Per-class diagnostic observations.

Product Class	Object Type	Main Visual Challenge	Diagnostic Observation
18 + 0.45 L	Bottle	Large object; moderate reflection	Stable detection and tracking in both views
Chortoq 0.5 L	Bottle	Similar bottle shape to other drinks	Occasional confusion under hand occlusion
Fanta 0.5 L	Bottle	Bright packaging; high visibility	Reliable detection and trajectory interpretation
Moxito 0.5 L	Bottle	Partial occlusion during removal	Continuous tracking improves recovery over snapshot comparison
Snickers 80 g	Small confectionery	Small pixel height; hand occlusion	Main source of remaining false negatives
Super Kontik 100 g	Small confectionery	Similar shelf position and size to Twix	Occasional confusion with Twix
Twix 75 g	Small confectionery	Similar packaging and size to Super Kontik	Sensitive to shelf re-settling and short visibility

**Table 13:** Runtime breakdown under the selected-frame transaction-processing protocol on Intel N100 CPU hardware.

Stage	Wall Time Per Transaction (ms)	Percentage of Total
YOLO11-seg inference on selected frames	241	42.9%
RT-DETR inference on selected frames	221	39.3%
Frame preprocessing, detection merging, and NMS	78	13.9%
ByteTrack association	12	2.1%
Zone classifier, FSM, and evidence-gate fusion	3	0.5%
Logging and receipt construction	7	1.2%
Total	562	100.0%

**Table 14:** Contextual comparison with adjacent retail vision systems.

Approach Type	Main Sensing Input	Temporal Reasoning	Multi-Camera Fusion	Billing Interpretability	Main Limitation
Static product recognition	Single image or isolated frame	No	No	Low	Cannot infer taken, returned, or inspected events
Snapshot-difference checkout	Start/end frames	No	Optional	Moderate	Blind to intermediate product motion
Generic object tracking	Video stream	Yes	Usually no	Moderate	Produces tracks but not receipt-level events
Learned video classifier	Video sequence	Implicit	Possible	Low	Limited auditability of decisions
Stereo/depth-based sensing	Calibrated multi-view or depth input	Possible	Geometric fusion	Moderate	Requires calibration, synchronization, or depth hardware
RFID/weight-assisted checkout	RFID tags, weight sensors, shelf load cells	Not primarily visual	Sensor-level fusion	High	Requires additional hardware and maintenance
Proposed ZAB-Fusion	Two asynchronous camera sensors only	Explicit zone-state reasoning	Evidence-gated event-level fusion	High	Evaluated on one cabinet and one deployment site

## Data Availability

The transaction videos used in this study are not publicly available because they were collected from a real in-service smart vending deployment and may contain incidental customer interactions and commercially sensitive operational information. To protect privacy and deployment confidentiality, raw videos cannot be shared publicly. Aggregated numerical results, derived non-identifiable statistics, and selected anonymized examples may be made available from the corresponding author upon reasonable request, subject to permission from the deployment operator and applicable privacy restrictions.
